# A comparison of metrics for quantifying cranial suture complexity

**DOI:** 10.1098/rsif.2020.0476

**Published:** 2020-10-07

**Authors:** Heather E. White, Julien Clavel, Abigail S. Tucker, Anjali Goswami

**Affiliations:** 1 Department of Life Sciences, Natural History Museum, London SW7 5BD, UK; 2 Centre for Craniofacial and Regenerative Biology, King's College London SE1 9RT, UK; 3 Division of Biosciences, University College London WC1E 6DE, UK; 4 Univ Lyon, Université Claude Bernard Lyon 1, CNRS, ENTPE, UMR 5023 LEHNA, F-69622, Villeurbanne, France

**Keywords:** suture, complexity, mammal, morphology, morphometrics, skull

## Abstract

Cranial sutures play critical roles in facilitating postnatal skull development and function. The diversity of function is reflected in the highly variable suture morphology and complexity. Suture complexity has seldom been studied, resulting in little consensus on the most appropriate approach for comparative, quantitative analyses. Here, we provide the first comprehensive comparison of current approaches for quantifying suture morphology, using a wide range of two-dimensional suture outlines across extinct and extant mammals (*n* = 79). Five complexity metrics (sinuosity index (SI), suture complexity index (SCI), fractal dimension (FD) box counting, FD madogram and a windowed short-time Fourier transform with power spectrum density (PSD) calculation) were compared with each other and with the shape variation in the dataset. Analyses of suture shape demonstrate that the primary axis of variation captured attributes other than complexity, supporting the use of a complexity metric over raw shape data for sutural complexity analyses. Each approach captured different aspects of complexity. PSD successfully discriminates different sutural features, such as looping patterns and interdigitation amplitude and number, while SCI best-captured variation in interdigitation number alone. Therefore, future studies should consider the relevant attributes for their question when selecting a metric for comparative analysis of suture variation, function and evolution.

## 1. Background

The quantitative analysis of shape is a well-established approach to robustly address questions across a breadth of disciplines and subdisciplines in biology, with broad application in the study of evolution, ecomorphology, development, biogeography, taxonomy and phylogenetics [1–10]. The ability to capture complex shape data has been greatly expanded by advances in geometric morphometric techniques which improve upon traditional morphometrics by providing the ability to capture information about where the parts of the shape are located with respect to each other in a Cartesian plane. These techniques have grown a particular interest and have been successful in the study of vertebrate morphology during the past three decades. Within the system of the vertebrate skull, the overwhelming focus of morphometric studies has been the bony structures themselves, with little attention given to the sutural joints between these cranial bones [11–22].

As sites of intramembranous bone growth, sutures are actively responsible for determining this cranial morphology via the facilitation of postnatal craniofacial development [23]. An intrinsic relationship clearly exists between sutures and cranial morphology, primarily due to sutures providing the principal growth site, although the relationship is not restricted to this and extends to other critical functional roles of sutures; facilitating brain development by allowing adequate space for brain expansion, fusing for protection of the developed brain, providing shock absorption during locomotion and facilitating feeding by providing joint mobility [24–26]. These suture functions have a clear impact on the cranial morphology. Despite their functional importance, suture morphology has seldom been studied in a comparative, quantitative framework and is considerably underrepresented in comparison to the well-studied morphology of the cranium. This stems from the lack of well-defined homologous anatomical landmarks for non-osseous and essentially featureless structures [27] while the open outline nature of sutures further adds to the inherent difficulty in quantifying their shape [28,29].

Suture morphology is known to be highly variable from suture to suture, across development, between the sexes and across species. When comparing suture to suture, those in the facial region have been reported to have straight morphologies compared to the interdigitated sutures of the braincase [30]. Looking across development, sutures have been suggested to progress from straight to highly interdigitated morphologies [31]. Sexual dimorphism of sutures has also been identified, with males exhibiting greater levels of complexity for facial sutures, thought to reflect the pressures of head-to-head fighting [32]. Interspecific species variation has been described at different levels, both within genus (*Cebus*) and within infraorder (Caviomorpha), where suture morphology ranges from low to high suture interdigitations [33,34]. Beyond this level, very little work has been conducted that addresses interspecific variation across larger clades, although it has been postulated that mammals have more intricate and interdigitated sutures than reptiles [30]. Evidently, a huge degree of suture variation has been observed at multiple levels of comparison.

The few papers that have quantified this variation in suture morphology to date, have suggested a wide range of drivers shaping suture morphological variation including innovation over evolutionary time, novel biomechanical relationships to ecological variation, mechanical strain from compressive forces, patterns of growth and development, and responses to environmental stress [29,30,35–38]. More specifically, the aspects of complexity shaping this morphological variation have been associated with a number of biological pressures. These biological pressures create heightened biomechanical stress across the skull, with the resultant effect being a deformation of the sutures to an interdigitated morphology [39]. Interdigitations act as shock absorbers by increasing the surface area for collagen and compression-resisting fibre attachments to dissipate the strain experienced by surrounding bones [39,40]. Biomechanical stress shaping this interdigitated suture morphology is thought to be emergent from a number of biological inputs: head-to-head fighting, tougher foods, chisel tooth digging, jaw opening and closing during mastication, and masticatory muscle mass [30,33,34,40–42]. However, very little is known about the forces generated from these sources of biomechanical stress and the subsequent impact on suture morphology [43]. Unmistakeably interdigitations play a huge part in the biological relevance of suture morphology. However, as both amplitude and frequency form this interdigitated morphology [29], it is unclear from the current studies whether one or both of these aspects of interdigitation are functionally relevant to the suture and thus biologically speaking important. Nevertheless, the suture morphological variation appears to reflect adaptations to mechanical demands from the environment, habitat and ecological pressures. The wider significance of how these pressures and biomechanical stresses impact suture morphology is currently limited by the absence of comparative studies in a phylogenetic framework which are necessary to place the biological interpretations into a broader evolutionary context [37].

Variation in suture morphology has been captured in a range of biological systems, from the vertebrate cranium to the ammonoid shell using methods that attempt to quantify complexity [28–30,33,34,37,44]. The implementation of a complexity metric, in contrast with using shape data, such as semi-landmarks, offers the advantage of generating a single quantitative value that provides the ability to easily make direct comparisons across species, suture locales and developmental ages. Moreover, shape incorporates many other attributes beyond complexity, complicating functional interpretations across diverse systems. For the purpose of our study, we consider complexity to measure how a suture deviates from its simplest form, a straight line, across its overall path. By contrast, shape captures additional aspects such as orientation and a reversal of the same morphology. While multiple methods have been developed to describe suture morphology as a complexity metric with this in mind, the transformation of suture morphology into meaningful quantitative data still does not come without its challenges. The intricate nature of sutures produces patterns that are hugely diverse even within an individual suture. Sutures reflect a tremendous degree of natural complexity, making their morphology inherently difficult to quantify. The challenges, however, are not limited to the suture morphology, but are further complicated by the methods themselves. The methods previously applied, operate on a variety of mathematical approaches, each of which adopts different assumptions, therefore resulting in variation for the definition and aspects of complexity captured. Moreover, many previous methods attempt to quantify morphology by means of a univariate statistical complexity value, whereby disparate morphologies have been reported to be present with near-identical complexity values, meaning it is impossible to reconstruct the original suture morphology [45]. An outline of the approaches and equations used here can be found in the electronic supplementary material (electronic supplementary material, text S1).

Despite the many methods proposed, there is no consensus regarding which approach is the most appropriate for capturing suture morphology as a quantified value of complexity, and in particular if the different approaches capture various aspects of the suture shape complexity which may be appropriate for addressing different biological questions. While we know that complexity in the form of an interdigitated shape has functional roles at the sutures, other aspects of complexity may also be functionally significant but have not been recognized in the limited previous work on this topic. Direct comparison of the strengths and weaknesses of the alternative approaches and identification of the different aspects of complexity each approach captures, will inform further research into suture morphology, development and evolution. In addition, the direct comparison of the approaches will offer the potential to integrate the results of studies implementing different complexity metrics. Here, we compare five methods for quantifying suture complexity: sinuosity index (SI), suture complexity index (SCI), fractal dimension (FD) box counting approach, FD madogram approach and windowed short-time Fourier transform (STFT) with a power spectrum density (PSD) calculation, using a dataset sampled across a diverse range of mammal suture morphologies. Mammal crania exhibit an incredible variety of shape and suture morphology, likely related to their complex mastication, and dietary and ecological diversity [46]. To accompany this cranial diversity, a wide range of suture morphologies are observable across mammals. Moreover, they are abundant and easily available in museum collections, thus providing the ideal sample dataset for comparing the metrics for quantifying suture morphology and complexity. Determining the aspects of complexity captured by the various metrics and which is the most appropriate approach for quantifying cranial suture complexity on this diverse empirical dataset will allow for future detailed study of the function, development and evolution of sutures across mammals.

## 2. Methods

### 2.1. Data collection

**Figure 1.**
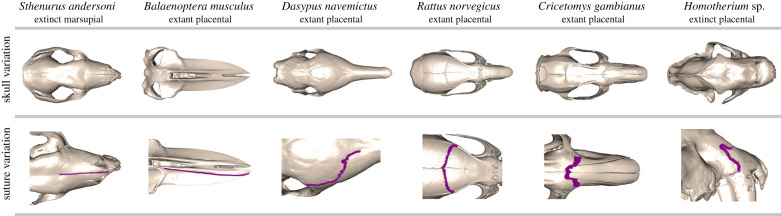
An example of skull and suture variation captured by the dataset from a broad range of mammalian taxa.

A sample of 79 extinct (*n* = 34) and extant (*n* = 45) mammals was generated to assess and test approaches available for quantifying suture morphological complexity (electronic supplementary material, table S1). Multiple sutures were sampled across this sample dataset rather than a single homologous suture, as the goal was to determine the most appropriate metric for a sample with a broad range of morphologies and not to assess the morphological variation within the sample dataset (figure 1). Spirit preserved specimens were scanned using the X-Tek HMX ST 225 µCT scanner (Nikon, Tokyo, Japan) and Go!Scan 20 (Creaform), Go!Scan 50 (Creaform) or EDGE ScanArm HD (FARO) scanners were used for osteological specimens. Scans were reconstructed in Avizo v.9.3 (FEI, Hillsboro, OR, USA), Geomagic Wrap (3D Systems), and VXElements v6.0 (Creaform) and cleaned in Geomagic Wrap leaving only the skull elements in the three-dimensional isosurface. Two-dimensional images (*n* = 79) of the multiple different sutures across the sample dataset were captured using uniform positioning to minimize the impact of parallax in the *rgl* R package [47]. Two-dimensional semi-landmarks were manually positioned on the suture images using the *StereoMorph* R package [48] and resampled at 500 semi-landmarks per suture to capture the open suture outline. Studies should first determine the number of manual landmarks required to capture the entire suture curve profile and resample to a number of landmarks that does not risk losing morphological complexity. This is particularly important as fractal-like sutures could be more sensitive to the number of landmarks positioned. Generalized Procrustes analysis was performed on the resampled two-dimensional semi-landmarks to remove all non-shape elements using the ‘*gpagen*' function in the *geomorph* R package [49,50]. Using the two-dimensional superimposed semi-landmarks, a principal component analysis (PCA) was performed to determine the major axes of shape variation captured by the sample dataset and whether these reflected variations in complexity.

### 2.2. Complexity analysis

**Figure 2.**
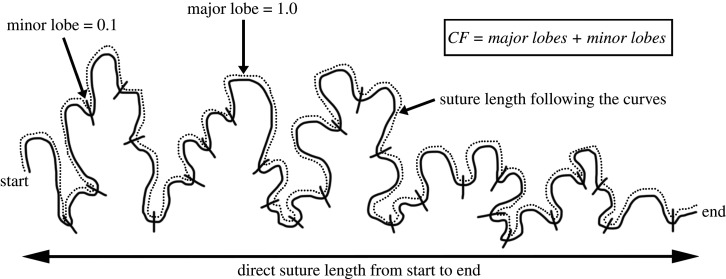
Depiction of SI and SCI calculation [51].

The five complexity metrics were applied to the Procrustes superimposed two-dimensional semi-landmarks. SI calculations were implemented in base R (v.3.6.0; R Core Team, 2019) using a standard distance between two points equation (electronic supplementary material, textS2: equation 7) in order complete the SI equation (electronic supplementary material textS2: equation 1, figure 2) [51]. SI has also previously been referred to as total sutural interdigitation [52] and relative suture length [33]. SCI used the SI value calculated in base R (v.3.6.0; R Core Team, 2019) and a complexity factor multiplier which was calculated from the interdigitation lobes (major and minor) [53] (figure 2). FD was calculated in the *fractaldim* R package [54], using both the box counting and madogram methods by implementing the ‘*fd.estim.boxcount'* and ‘*fd.estim.madogram*' functions, respectively. STFT was computed using the ‘*stft*' function in the *e1071* R package [55]. The PSD of each suture was calculated using the STFT results, by averaging the squared STFT coefficients over each frequency across the local transforms and summing the averages at each harmonic, as described by Allen (2006) [29].

### 2.3. Comparative analysis of the complexity metrics

The appropriateness and effectivity of each metric was assessed using multiple comparative approaches. Comparisons between suture shape data and complexity scores were made to determine whether the metrics captured functionally relevant suture morphologies. Functionally relevant aspects of suture morphology include, but are not limited to, the multiple features of interdigitations (number and amplitude) thought to be shaped by biomechanical stresses as a result of behaviour and diet. Correspondence between complexity score and suture shape PC score were quantified with Pearson's correlation coefficient (r) and visualized with a heatmap using the ‘*corrplot*' function from the *corrplot* R package [56]. The significance (*p* < 0.05) of the correlations were tested using the ‘*cor.mtest*' function [54]. A heatmap reflecting complexity scores for each specimen was mapped onto the PCA of Procrustes superimposed two-dimensional semi-landmarks to further determine whether the complexity metrics captured the functionally relevant aspects of morphological variation. Similarly, correlations between the five complexity metrics were quantified and visualized using the ‘*corrplot*' function in the *corrplot* R package, significance was tested using the ‘*cor.mtest*' function [56]. Finally, we conducted a further PCA on the specimen complexity scores, rather than the shape data, using the *factoextra* R package [57]. The contribution of each variable (*n* = 5; complexity metrics) on the PC axes (i.e. the scaled PC loadings) were plotted on a correlation circle. The PC loadings were superimposed onto the PCA, of the first two axes, thus representing both variables (*n* = 5) and individuals (*n* = 79) using the ‘*fviz_pca_biplot*' function, in order to assess how the approaches, relate to each other and the complexity across the sample dataset. Further detail on the methodology is provided in the electronic supplementary material (electronic supplementary material, text S2). The datasets generated and/or analysed throughout our study are available in the Github repository: https://github.com/HeatherEWhite/suture_metrics_comparison [58].

## 3. Results

### 3.1. Shape analysis

**Figure 3.**
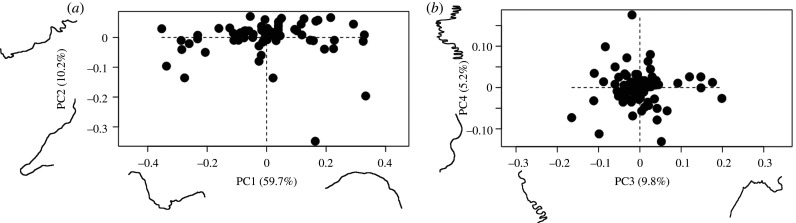
Principal components analysis of two-dimensional semi-landmark shape data with extreme suture morphologies for: (*a*) PC1 and PC2; (*b*) PC3 and PC4.

Shape analysis refers to variation in the shape variables themselves, the Procrustes superimposed semi-landmarks. PCA of the Procrustes superimposed two-dimensional semi-landmarks (figure 3) identified 19 principal components (PCs) that explained 99% of the shape variation (electronic supplementary material, table S2). Of these PCs, the first four each accounted for greater than 5% of the overall variation, with PC1, PC2, PC3 and PC4 accounting for 59.7%, 10.2%, 9.8% and 5.2%, respectively. The main axis of variation (PC1) was associated with an inversion of the suture morphology, with the positive aspect of PC1 reflecting an ‘n' shaped morphology and the negative aspect reflecting a ‘u' shaped morphology (figure 3 and electronic supplementary material, figure S2). Variation along PC2, PC3 and PC4 more clearly reflected differences in the complexity of shape, such as looping patterns, irregularity and straight to interdigitated sutures (figure 3 and electronic supplementary material, figure S2).

### 3.2. Complexity analysis

**Figure 4.**
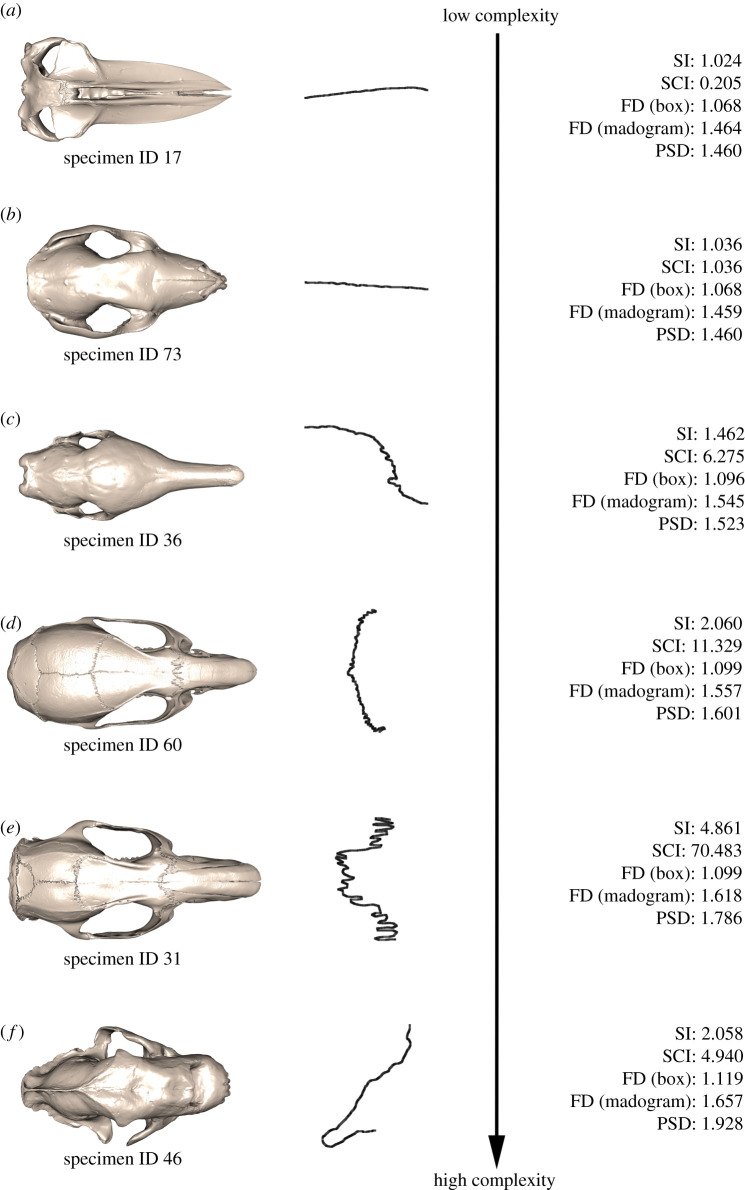
Suture complexity variation from low (*a*) to high (*f*) complexity within the mammal skull sample (*n* = 79). Results presented for all five methods tested: SI, SCI, FD box counting, FD madogram, PSD. Specimen ID and their species associations are detailed in electronic supplementary material, table S1.

Complexity analysis captures variation in the complexity scores across the dataset, which are estimated from the suture shapes, but not the shapes themselves. The five methods produced different ranges of complexity across the sample dataset, with the largest value indicative of the most complex suture: 1.024 to 4.861 for SI; 0.205 to 87.963 for SCI; 1.055 to 1.120 for FD (box counting method); 1.452 to 1.657 for FD (madogram method); 1.449 to 1.928 for PSD, extreme morphologies for each metric are presented in electronic supplementary material, figure S3. Full results for each suture are presented in electronic supplementary material, table S3, with a subset of sutures representative of the range of suture complexity detailed further in figure 4.

Sutures recognized as ‘simple' (figure 4*a,b*) following SI, SCI, FD (box counting), FD (madogram) and PSD analysis presented with a largely straight morphology and very few interdigitations. For each of the simple sutures, all metrics indicated a low complexity value. The FD and PSD complexity values were very similar to one another, with greater differences observed for the SI, SCI and FD (box counting) metrics.

Sutures recognized as ‘moderately complex' (figure 4*c*,*d*) by all the applied metrics presented with a morphology that deviated away from a straight line accompanied by a greater number of interdigitations that were small in magnitude. All methods quantified these morphologies with higher values than for the simple sutures.

The ‘most complex' sutures, recognized by the majority of applied metrics, exhibited large magnitude interdigitations, irregular repetitions and looping patterns (figure 4*e*,*f*). These more complex sutures had higher SI values than the moderately complex sutures. SCI indicated that suture 31 (specimen ID 31), which had interdigitations of a heightened magnitude, was highly complex compared to the relatively simple suture 46 (specimen ID 46), which presented with an irregular looping pattern. PSD, however, showed the opposite, with suture 46 (specimen ID 46) being the most complex of the two, although suture 31 (specimen ID 31) was still represented as complex. FD box counting and FD madogram values returned very similar values for the two sutures considering them both to be similarly highly complex sutures. Evidently, while both sutures were identified as complex by the majority of metrics their morphologies were largely disparate. These differences in morphology appeared to be better reflected by the SCI and PSD values, as these both ascribed quite different complexity scores to the two sutures compared to the values determined by SI, FD box counting and FD madogram. Scenarios, where the five complexity metrics disagree, undoubtedly become more evident with the more highly complex sutures as a result of greater shape variety in the more complex sutures which is not always limited to interdigitations.

### 3.3. Comparative analysis of the metrics

**Figure 5.**
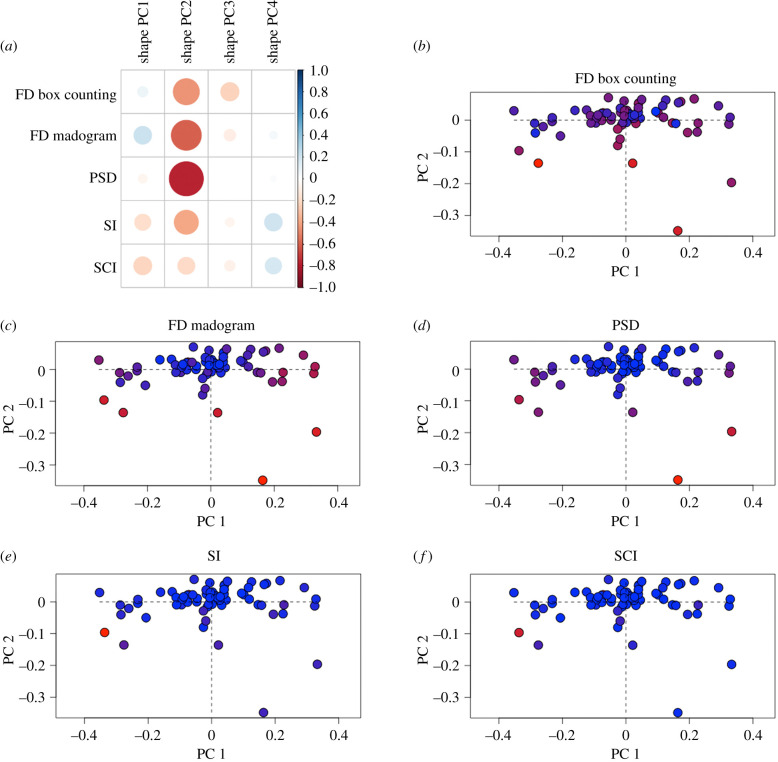
Principal components analysis of two-dimensional semi-landmark data and its relationship to the complexity methods: (*a*) correlation plot between the principal component scores of the shape data and the complexity scores from the five complexity metrics; (*b–f*) PCA of shape data with heatmaps indicating the complexity scores for FD box counting, FD madogram, PSD, SI and SCI complexity scores, respectively. Complexity scores for each of the five methods were subdivided into 10 equal bins to define the heatmap colour associations, with red indicating the most complex sutures and blue indicating the least complex sutures.

Here, we consider how variation in shape compares to variation in complexity, while addressing the relationships between each complexity metric. PC scores for all specimens were extracted from the PCA of suture shape variation (figure 3), for the PCs that explained greater than 5% of the overall shape variation (PC1, PC2, PC3 and PC4) (electronic supplementary material, table S4). Correlation analysis between these PC scores and the method complexity scores shows that PC2 aligns most strongly with all of the complexity metrics, except SCI (figure 5, electronic supplementary material, table S5). Of the five implemented complexity metrics, PSD has the strongest correlation to PC2 (*r* = −0.766, *p* < 0.001). When the specimen complexity scores are mapped onto the shape morphospace using heatmaps, marked differences across the different metrics relative to suture shape are evident. Both SI and SCI returned relatively few highly complex sutures (figure 5*b*,*c*), but these always fell in the negative region of PC1 and PC2. For FD box counting, FD madogram and PSD metrics the most complex sutures clustered in the negative region of PC2, while the least complex sutures appeared at the positive end of PC2. By contrast, there was little association between complexity scores and the shape variation described by PC1 for these three metrics. Complex sutures were present on either end of PC1 for both FD madogram and PSD, whereas for FD box counting, it was the sutures with lower complexity that appeared at both extremes of the PC1 axis. PCA of the two-dimensional semi-landmarks using heatmaps to indicate complexity (figure 5*b–f*), support the correlation analyses (figure 5*a*) that indicated the complexity metrics were most strongly correlated with the shape variation of PC2.

**Figure 6.**
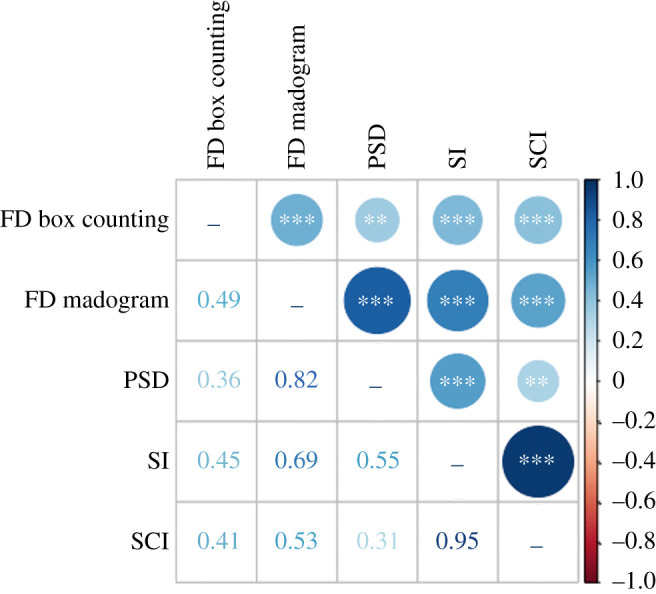
Correlation plot comparing complexity metrics indicating in the lower half the correlation coefficients (r) and in the upper half the strength of the correlation by colour and circle size, * *p* < 0.05, ** *p* < 0.01, *** *p* < 0.001.

Direct comparisons across the five implemented complexity metrics indicated that there were very few strong correlations (*r* > 0.7) (figure 6*a*, electronic supplementary material, table S6). The strongest correlation was unsurprisingly between SI and SCI (*r* = 0.954, *p* < 0.001), as SCI is a modification of SI. Interestingly, there was a further strong correlation between the FD madogram method and PSD method (*r* = 0.823, *p* < 0.001). This correlation was much stronger than the correlation between the two FD metrics even though a similar fractal method underlies both of the FD approaches, suggesting the two fractal metrics are largely different thus capturing variations in the suture shape complexity. Furthermore, the 95% confidence intervals for these two comparisons (SI versus SCI and FD madogram versus PSD) were small and thus emphasized a strong correlation. Only moderate correlations (0.3 < *r* < 0.7) were identified for all other complexity metric comparisons, with correlation coefficients ranging between 0.314 and 0.689. These moderate correlation coefficients presented with a wider 95% confidence interval than the stronger correlations, emphasizing uncertainty in estimating the correlation coefficient between the majority of metrics. All results were statistically significant (*p* < 0.01) (figure 6*b*).

**Figure 7.**
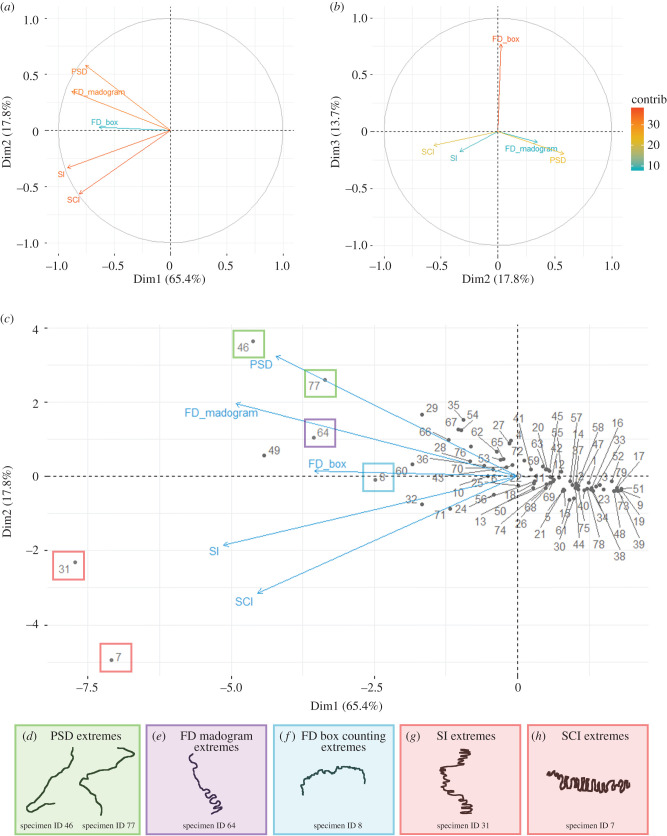
Principal components analysis of suture complexity, with variables reflecting the five complexity metrics (SI, SCI, FD box counting, FD madogram, PSD): (*a*) variable correlation plot for PC1 and PC2; (*b*) variable correlation plot for PC2 and PC3; (*c*) PCA for PC1 and PC2, plotted with the individual specimens (PC scores); (*d-h*) PSD, FD madogram, FD box counting, SI and SCI morphological complexity extremes, respectively. Specimen ID and their species associations are detailed in electronic supplementary material, table S1.

PCA performed using the method complexity scores and plotted with each metric as a variable (figure 5) indicated that the five metrics captured a different aspect of complexity, evidenced by the diverging variables which reflect opposite PC loadings on the first components (figure 7*a*,*b*). The first three PCs explained 95% of the overall complexity variation (electronic supplementary material, table S7), with the first PC accounting for 65% of the variation (figure 7*a*). The loadings (i.e. the correlations between the PC scores and the complexity data) of the five metrics on PC1 are all positively related and show that this major axis of variation mostly explains the range of values from the five metrics, with the least complex sutures at the positive aspect and more complex sutures at the negative aspect (figure 7*c*; electronic supplementary material, figure S4*a*). However, the loadings on PC2 (17.8%) show contrasting correlation patterns between the metrics on this axis, seen by the diverging variables (figure 7*a*). Specifically, SI and SCI are loaded together towards the negative aspect of PC2, whereas FD madogram and PSD are associated in the opposite direction of PC2. A strong correlation between the variation of PC2 and the SI, SCI, FD madogram and PSD methods was identified from the four variables approaching the outer circle limit. Of these loadings, PSD and SCI contribute the most to the overall variation in PC2 (electronic supplementary material, figure S4*b*). By contrast, FD box counting accounted for very little of the variation explained by PC2 but is instead highly loaded and aligns best with PC3, which explains 13.7% of the overall variation (figure 7*b*, electronic supplementary material, figure S4*c*). No other PC axis explained ≥5% of the variance in the complexity metrics.

The opposite association between the complexity metrics along PC2, from the PCA of suture complexity (figure 7*c*), indicate that suture morphological complexity is multi-faceted, whereby each of the various metrics are effective at capturing different characteristics of suture morphological complexity. Individual specimen complexity scores plotted onto this PCA (figure 7*c*) show the extreme morphological complexities captured by the various metrics. Specimen ID 7 (figure 7*h*) reflects the extreme morphological complexity of SCI characterized by numerous interdigitations along the suture length with a heightened magnitude and a slight irregular pattern. Specimen ID 31 (figure 7*g*) highlights the extreme of SI morphological complexity characterised by a regular repeating pattern, with numerous interdigitations of high magnitude. Specimen ID 8 (figure 7*f*) reflects the extreme morphological complexity of the FD box counting method and is characterized by a regular patterning, with many small interdigitations. Specimen ID 64 (figure 7*e*) reflects the extreme morphological complexity of the FD madogram method, characterized by an irregular pattern with a heightened number of high magnitude interdigitations captured by FD madogram complexity. Specimen IDs 46 and 77 (figure 7*d*) represent the extreme morphological complexity of the PSD method, whereby PSD perceives morphological complexity as a largely irregular pattern, with folds of high magnitude and a looping morphology. Furthermore, along PC2 where the methods diverge, the spread of the specimens is positively associated to the FD madogram and PSD method and negatively associated to the SI and SCI methods.

## 4. Discussion

Sutures play a critical role in the development and functioning of the skull throughout an organism's life [23–26,59–61], and thus suture morphology is important for understanding skull morphology and function. Complexity, in particular, has been discussed as a critical variable for skull function [30], but approaches that quantify complexity and their relationship to suture shape have not been directly compared. We tested and compared five complexity metrics (SI, SCI, FD box counting, FD madogram and PSD) based on different mathematical approaches for quantifying suture complexity across a range of mammalian cranial suture morphologies. While there was some correspondence across methods, they each captured different aspects of suture morphological complexity, with PSD showing a particularly strong correlation with PC2, which captured diverse aspects of complexity represented in this sample dataset. Therefore, studies should consider which metric, or a combination of metrics is most suitable depending on the aspect of suture complexity that is of interest.

### 4.1. A complexity metric could be more useful than raw shape data for studying sutures

The complexity metrics were most strongly correlated with the shape variation of PC2, rather than PC1 which accounted for approximately 60% of the overall shape variation. The shape variation of PC1, from the PCA of two-dimensional semi-landmarks, captured an inversion of shape or mirror image (‘n' versus ‘u' shaped morphology at the opposite extremes of PC1 on figure 3*a*) and did not appear to capture functionally relevant variation in complexity, such as looping patterns, straight morphologies and interdigitations. The largest variation in shape complexity, rather than a reversal of the same shape, was captured by PC2. As nearly all of the complexity metrics correlated most strongly with this axis of variation, it is clear that the complexity metrics are capable of capturing the predominant variation in morphological complexity.

Indeed, the complexity metrics are only extracting part of the suture shape information, but these metrics are most highly correlated with the features of suture shape that have the highest functional relevance to the suture. This suggests that using the raw shape data alone to understand sutural complexity and morphological variation could be misleading and instead supports the use of a complexity score. The use of a complexity score which provides a single value is a useful tool to make direct comparisons across diverse datasets. Attempts to use complexity scores to make comparisons of suture complexity across different species from various published studies has previously been attempted [30,37]. However, these studies have been performed on various taxa using various methods, thus with little overlap, meaning comparative data are sparse despite the consensus to evaluate complexity based on interdigitations. Therefore, determining a single complexity metric, as our study aimed to do, will prove incredibly useful to make such comparisons. Moreover, the comparisons made by our study of the previously implemented complexity metrics, identify which aspects suture shape each approach captures. This will further prove useful for future studies, to aid in the determination of the most appropriate metric dependent on the biological questions and empirical dataset proposed by the study.

### 4.2. Different complexity metrics capture different sutural features

Comparing the distributions of complexity scores for the five metrics and their correlations with each other and with suture shape allows us to identify which aspects of suture complexity each approach captures. PSD greatly contributed to the major axes of complexity score variation and therefore describes a very high level of variability in suture complexity across a range of mammal suture morphologies. Moreover, from the PCA analysis of raw shape data, PSD correlated most strongly with PC2, which largely reflected variation in suture complexity rather than a mirroring of the suture shape, as captured by PC1 (figure 3*a*). While the other metrics also correlated strongly with PC2, PSD appeared to be more sensitive to differences in suture shapes, such as looping, at higher end of complexity scores. As a result, we observed that PSD discriminates among disparate morphologies that were scored as equally complex by non-Fourier methods (FD box counting and FD madogram). This difference between PSD and FD approaches, shown here with mammal cranial sutures, supports previous analysis of ammonoid sutures where a difference between SCI and PSD was also identified [29,45,62]. Therefore, PSD may be the most appropriate metric when diverse aspects of complexity are present, as in mammal cranial sutures. It may be unsurprising that our analyses identified PSD as the most appropriate method for quantifying suture complexity, given that the method is based on a windowed STFT approach [29,45]. The Fourier foundation of PSD suggests that this approach is mathematically robust, as the statistical transformations, such as windowing techniques, account for profiles in nature, such as suture morphology, that are discrete and non-stationary [63]. Therefore, PSD provides a mathematical description of the suture morphology, by implementing a Fourier transform approach, that is unique to each suture morphology [45].

Of the two FD methods implemented (box counting and madogram), it was perhaps unsurprising that the madogram method most closely followed the loadings of the PSD variable, given its reported efficiency and robustness over other FD methods, including the box counting and ruler dimension methods [64]. Interestingly, the FD ruler dimension approach is more commonly applied to suture complexity data [30,37,42] than the FD box counting approach [65], despite it having been argued that such an approach should not be implemented on observational data [63]. Despite its robustness, the FD madogram method has not been previously applied to the suture complexity problem. As FD madogram has been previously identified to be the most robust of the FD methods, this supports our findings of a stronger correlation between FD madogram and PSD, than the FD box counting approach and PSD. However, it was surprising that the stronger correlation was observed between the FD madogram and PSD metrics than between the two FD metrics which are based upon very similar fractal methods.

The FD box counting metric mapped well to both the features reflected by PC2 and PC3 of shape variation. Of the approaches, this metric appears least sensitive to the extreme forms sampled within the dataset thus producing well-behaved results. However, it has been described as less robust and efficient than the FD madogram metric [64]. There is, however, a critical issue in the use of fractal analysis for patterns in nature, such as suture morphology, because natural forms are well documented to be intricate and often do not follow the self-similarity assumptions of fractal analysis [29]. More specifically, serrated sutures generally do not follow self-similarity but can be very intricate. Of the FD approaches, FD madogram has been suggested to align better with the self-similarity assumptions [63] suggesting a potential advantage over the other FD methods.

Regardless of the issues with the FD methods, previous studies still identified FD as a better objective descriptor of suture complexity than SCI [65]. While SCI calculates complexity based on a length ratio measurement (SI), it also includes a subjective and arbitrary measure of interdigitation number and indentation. This likely results in the insensitivity we observed to other aspects of geometric complexity, which is also an issue for the SI approach that provides the foundation for SCI. The resultant effect of a linear length measurement for complexity is that shallower lobes would give similar complexity values to fewer deeper lobes [53] in contrast to PSD. Nevertheless, SCI is effective at determining complexity based on interdigitation number, due to its inclusion of a complexity factor multiplier [53]. This disparity between SCI and PSD was also identified in a previous study [29] comparing SCI and PSD for ammonoid suture morphology.

The lack of very strong correlations among the tested methods indicates that the five methods capture different aspects of suture morphological complexity. Suture morphology is hugely diverse from straight to incredibly intricate morphologies, each with varying complexity scores. Of the features forming suture morphological complexity, previous work has implied that interdigitations are the most biologically relevant feature of a suture form. This is because interdigitations are thought to reflect the biomechanical stress associated with diet [30,33,34,42], extrinsic mechanical factors [40,43,59,66,67] and also suture development [68]. From the PCA analysis of complexity, SI and SCI were most strongly associated with the interdigitated morphologies. For these methods, the more complex sutures were those with a greater number of interdigitations.

Interdigitation complexity is not, however, defined simply as the number of interdigitations. The SI and SCI methods focus on this one aspect and disregard the other features that form these interdigitations, such as amplitude. While SI and SCI captured well the number of interdigitations, PSD offered the ability to capture both heightened interdigitation number and greater interdigitation amplitude as complex [29]. FD box counting and FD madogram also captured interdigitation magnitude to a greater extent than SI and SCI, as well as capturing interdigitation number. Therefore, while all approaches are able to account for the interdigitations formed as a result of biomechanical stress from external inputs including diet and behaviour [33,40], PSD and both FD metrics likely better capture the details interdigitations are comprised of. As interdigitation is thought to reflect biomechanical stress produced from a number of biological pressures [30,33,34,39–42], it is likely that complexity score reflects skull function. More specifically, sutures exhibiting a higher complexity score are likely to possess a greater ability to absorb stress from external dietary and behavioural inputs. Moreover, we expect that a greater degree of complexity may accumulate with developmental age, as functional pressures on skulls increase. Beyond interdigitations, further aspects of complexity, such as irregularity, are incorporated into the PSD method definition of complexity [29] but have been seldom discussed in previous studies. It is possible that these previously disregarded additional aspects of complexity are biologically important or entirely irrelevant to the suture functioning, but this is currently unknown.

Each metric has strengths and weaknesses relative to different aspects of suture complexity. Therefore, in future studies, the appropriate metric should be determined based on the questions posed. For the sample dataset of mammalian cranial sutures, spanning a range of morphologies, used in our study, we prefer PSD, due to its ability to discriminate between disparate morphologies based on multiple aspects of complexity. However, a combination of metrics could also prove useful, such as PSD and FD box counting, to ensure the range of suture morphologies across the major axes of complexity variation (PC2 and PC3) are captured. Understanding the features of suture complexity captured by each of the methods will assist in making direct comparisons across the literature where various approaches have previously been employed.

While we found PSD to be a promising metric for comparative quantitative analysis of suture morphological complexity, we only compared mammalian taxa, and thus other methods may prove more appropriate for other systems. Nevertheless, we have no reason to believe that the PSD method would be unsuccessful for other taxa, in particular since it has been previously identified as a useful method for ammonoid sutures [29]. Furthermore, this study compared sutures covering a broad range of morphologies and thus may not be as powerful for detecting minor differences in sutures, such as those that may be encountered in an intraspecific dataset for a single suture, depending on the attributes of interest. From our analysis, it appears as though the sampled percentage of the sutural segment plays a role in determining the suture complexity score. Different regions of sutures may display different complexities. For example, sampling a smaller segment of suture 46 (specimen ID 46) would likely generate a lower complexity score. Therefore, it is imperative that future studies consider and are consistent with the scale of sutural complexity analysis by separating out macro-/meso-/micro-scale suture sampling. Moreover, our analysis is currently limited to two-dimensional data, due to the currently available implementations of each method. In many systems, analysis of two-dimensional data closely approximates that of three-dimensional data [69], but it is not possible to assess this for suture morphology at present. It is hoped that the recent advances in three-dimensional geometric morphometrics [21,70–72] will quickly broaden the quantification of sutures in three dimensions. When this becomes possible, it will be important to compare the results of two-dimensional and three-dimensional studies of suture morphology and assess whether the same methods work well with both approaches, as has occurred in recent years with studies of skull and body shape [73–75]. Given that sutures provide the main joints of the skull, consideration of their three-dimensional complexity could be crucial in understanding their overall functioning. Despite our use of X-ray microtomography and laser surface scanning to produce the dataset, all five complexity metrics could equally be implemented on high-quality (two-dimensional) photographs. When implementing the metrics on two-dimensional datasets, it would be critical to consider the possible effects of parallax. As a result of this two-dimensional implementation, each of the compared complexity metrics can be easily applied to a wide range of datasets and systems covering an array of scientific fields.

Finally, while many of the methods used here have been previously applied to quantifying suture complexity in a limited number of taxa, including caimans, primates, caviomorph rodents and ammonoids [30,65,76], this is the first study to apply and compare these methods across a wide range of extant and extinct mammal species and a breadth of suture morphologies. It is surprising that there have been so few prior quantitative studies of suture morphology in mammals, given the huge diversity of the group in terms of cranial morphology, diet, habitat and life history, all of which are known to generate variety in suture morphology [34,77,78]. Moreover, mammals are extremely well studied in terms of quantification of cranial morphology [79–84], cranial development [85–87] and cranial mechanics [88,89]. Therefore, the work presented here will be instrumental in bringing the quantitative analysis of suture morphology into the already rich field of mammalian cranial evolution. Our future work will apply the results of this study to a large-scale analysis of suture development and evolution across mammals.

## 5. Conclusion

Suture morphology has received relatively little attention in comparative biological studies, in comparison to the abundance of work focused on cranial morphology. While it has been well recognized that the quantification of cranial morphology with geometric morphometrics can offer invaluable insights into evolutionary patterns, ecomorphology, development, taxonomy and phylogenetics. It is also known that suture development and morphology play active roles in facilitating postnatal cranial development, skull function, feeding and shock absorption during locomotion. Indeed, it is for all these functional reasons that ecomorphological reconstructions, taxonomy and phylogenetic reconstructions have been performed for the skull. Consequently, it is highly likely that shape analysis of sutures can offer important insights for reconstructing the evolution and development of the skull.

In this study, we have provided the first comprehensive comparison of the available methods for quantifying suture morphology. Our analyses found that windowed STFT with a PSD calculation best captured the multiple aspects of suture complexity observed across diverse mammalian taxa and suture forms. PSD captured all aspects that form interdigitations, which are biologically important as they are strongly associated with high biomechanical stresses. Sutures with high PSD scores likely possess a greater ability to absorb stress from external biological pressures, than those with lower PSD scores. Nevertheless, all the metrics studied here capture some key aspects of sutural complexity, while all providing a univariate score which can be easily implemented for comparisons across sutures and taxa. Therefore, future studies attempting to quantify suture complexity, should first outline which features of suture shape are most relevant to address the questions of the specific study, in order to determine the most appropriate complexity metric. Quantifying suture morphology with a complexity metric in a comparative phylogenetic framework has great potential for bringing the understudied field of suture morphology into the vibrant fields of evolutionary and developmental biology.

## Supplementary Material

Supplementary Background, Methods, Tables and Figures

## Supplementary Material

Semi-landmark File

## Data accessibility

The semi-landmark data analysed during the current study are available in the electronic supplementary .csv file. Computer code and final raw data used during this study are available within a public GitHub repository at https://github.com/HeatherEWhite/suture_metrics_comparison [58]. All code and raw data are available to download, complete with MIT licence. Please cite this paper and the Zenodo doi: https://doi.org/10.5281/zenodo.4037220 when using the data or raw code.
