# Supplementary material for: A comparison of metrics for quantifying cranial suture complexity
Source: J R Soc Interface. 2020 Oct 7;17(171):20200476. doi: 10.1098/rsif.2020.0476 (PMC7653371; doi:10.1098/rsif.2020.0476)
Supplement: Supplementary Background, Methods, Tables and Figures [file rsif20200476supp1.pdf]

Supplemental Material

Text S1: Supplemental Background

Traditionally, methods applied to the suture complexity problem were based on length ratio measurements. Sinuosity Index (SI) (Equation 1) (Westerman, 1971) was the earliest method, followed soon after by Suture Complexity Index (SCI) (Equation 2) (Saunders, 1995), which utilised the foundation provided by SI but created an additional complexity factor (CF) to place a greater emphasis on interdigitations. Both SI and SCI utilise Euclidean geometry methods to generate a length ratio measure of complexity.

$$SI = \frac{suture\ length\ following\ the\ curves}{suture\ length\ start\ to\ end}$$ ***Equation 1***

$$SCI = SI \times CF$$

Where, SI = sinuosity index; CF = complexity factor ***Equation 2***

A revolution in the field of shape occurred following the pivotal work of Mandelbrot who developed fractal geometry (Mandelbrot, 1982) whereby shapes of expanding symmetry are produced from a series of iterations that appear remarkably like patterns in nature. Sutures form natural fractals, which led to the implementation of fractal dimension methods to quantify suture complexity as a statistical index that is widely applicable across diverse morphologies (Long, 1985; Boyajian & Lutz, 1992; Long & Long, 1992; Monteiro & Lessa, 2000; Skratz & Walocha, 2003). Specifically, fractal dimension measures the recurving of a suture. Sutures can be reflected as transect line data for the implementation of fractal dimension methods. One-dimensional methods of fractal dimension are reported to be suitable for transect line data, these include: box counting, Hall-Wood estimator, madogram estimator, variogram estimator, and spectral and wavelet estimator (Gneiting *et al.* 2012). Previous studies evaluating cranial suture complexity (Skrzat & Walocha, 2003) implemented the box count method (Equation 3). However, the madogram method (Equation 4) has since been recommended for use due to its higher efficiency and robustness (Gneiting *et al.* 2012).

$$\widehat{D}_{BC} = -\left\{\sum_{k=0}^{K}(s_k - \bar{s})logN(\varepsilon_k)\right\}\left\{\sum_{k=0}^{K}(s_k - \bar{s})^2\right\}^{-1}$$

where, N(ε) is the number of boxes required at width or scale ε; sk = log εk; $\bar{s}$ is the mean of s_0, $s_1,\ldots,s_K$ ***Equation 3***

$$\widehat{D}_{V;p} = 2 - \frac{1}{p}\left\{\sum_{l=1}^{L}(s_l - \bar{s})\log\widehat{V}_p\,(l/n)\right\} \cdot \left\{\sum_{l=1}^{L}(s_l - \bar{s})^2\right\}^{-1}$$

where, *p is* order of *p* of a stochastic process with stationary increments for the madogram approach *p* = 1; L ≥ 2; $s_l = \log(l/n)$; $\bar{s}$ is the mean of $s_l, \ldots, s_L$; $\widehat{V}_p(l/n)$ is the classical method of moments estimator ***Equation 4***

Given that sutures possess a huge degree of morphological complexity, it has been argued that previous methods utilising a single value of complexity are overly simplistic, produce ambiguous results, and are unable to distinguish between disparate morphologies (Lutz & Boyajian, 1995; Gildner, 2003). Quantifying sutures with periodic signals has been suggested to provide a more comprehensive description of suture complexity and morphology (Allen, 2006). Consequently, Fourier approaches, which summarise suture morphology as a series of sine and cosine functions (Gildner, 2003), have been suggested to form a stronger basis for studies considering morphological variation such as those utilising Elliptical Fourier Analysis (EFA) (Crampton, 1995; Tort, 2003; Carlo *et al.* 2011; Bardua *et al.* 2018, Emmons *et al.* 2018). EFA methods, however, deal with closed outline shapes such as an entire bone circumference. As sutures largely present as open outlines they violate the basic assumptions of EFA (leaking and aliasing) (Allen, 2006). Instead, for open outlines, it is possible to apply a windowed short-time Fourier transform (STFT) approach, which utilises coefficients calculated from a discrete Fourier transform (Equation 5) (Gildner, 2003; Allen, 2006; Wu *et al.* 2007). STFT obtains a rigorous mathematical decomposition of the suture morphology which can then be summarised to a single point estimate with a power spectrum density analysis (PSD) (Equation 6) (Allen, 2006). However, Fourier methods have seldom been applied to suture morphological variation thus far.

$$F_{k,m} = \sum_{n=0}^{N-1} f(n)w(n-mS)e^{-\frac{i2\pi(n-mS)k}{N}}, \quad k = 0, 1, 2, \dots, N-1$$

where, S is the skip frame, $F_{k,m}$ is the Fourier coefficient of the kth harmonic in the mth frame (window) along the length of the original discretely sampled $f(n)$. ***Equation 5***

$$P_T = \sum_{k=0}^{N/2} P(k)$$

where, $P(k) = |F_k|^2$, the power of the kth harmonic. ***Equation 6***

Text S2: Supplemental Methods

Specimens

A sample of 79 specimens was generated to assess and test methods available for quantifying suture morphology. This test dataset was selected to sample a diverse range of suture morphologies and therefore encompassed a broad range of mammalian taxa, including extant eutherians (n=41), extant marsupials (n=4), extinct eutherians (n=31) and extinct marsupials (n=3) (electronic supplementary material: table S1). Multiple sutures were sampled across this sample dataset rather than a single homologous suture, as the goal was to determine the most appropriate metric for a sample with a broad range of morphologies and not to assess the morphological variation within the sample dataset. The dataset was comprised of specimens from global museum collections (Muséum National d'Histoire Naturelle [MNHN], American Museum of Natural History [AMNH], The Natural History Museum UK [NHMUK], Los Angeles County Museum [LACM], Museo Argentino de Ciencias Naturales [MACN], Yale Peabody Museum of Natural History [YPM], Museo de La Plata [MLP], Grant Museum Zoology, University of Kansas Natural History Museum [KU], Field Museum of Natural History [FMNH], Bell Museum of Natural History [UMN], Museum Victoria Melbourne [NMV], Centre for Biology and Management of Population Montpellier [CBGP], Western Australian Museum [WAM], Smithsonian National Museum of Natural History [USNM]).

Scan preparation

Spirit-preserved specimens from the NHMUK were scanned using the X-Tek HMX ST 225 μCT scanner (Nikon, Tokyo, Japan) at the NHMUK. Osteological specimens from MNHN, AMNH, NHMUK, LACM, MACN, YPM, MLP, Grant Museum, KU, FMNH, UMN, NMV and USNM were laser surface scanned using the Go!Scan 20 (Creaform), Go!Scan 50 (Creaform) or EDGE ScanArm HD (FARO) scanners. Micro-CT scans were reconstructed in Avizo v.9.3 (FEI, Hillsboro, OR, USA) by compiling 2D X-ray projection slices to create 3D isosurface models (.ply) using a Filtered Back Projection algorithm (Kak & Slaney, 1988). Surface scans were reconstructed in Geomagic Wrap (3D Systems) and VXElements v6.0 (Creaform). All CT and surface scans were subsequently cleaned and prepared in Geomagic Wrap, whereby preparation involved the removal of vertebrae and the mandible, leaving only the skull elements in the 3D isosurface model.

Three-dimensional isosurfaces (n=79) were loaded in the *rgl* R package (Adler & Murdoch, 2019), to capture 2D images of the sutures. A single homologous suture was not used across all specimens; instead, many different sutures exhibiting a range of morphologies were captured by the 2D images (figure 1), as the purpose of the study was to determine the most appropriate metric over a range of suture morphologies and complexities.

Morphometric data collection

Open suture outlines were obtained from the 2D suture images (n=79), by the manual positioning of semi-landmarks along the individual suture outline from start to end, using the *StereoMorph* R package (Olsen & Haber, 2018) (electronic supplementary material: figure S1). Two-dimensional semi-landmarks were subsequently resampled at 500 per suture to ensure that suture complexity was accurately captured.

Shape analysis

To remove all non-shape elements, specifically rotation, translation, and isometric size (Rohlf & Slice, 1990) from the resampled 2D semi-landmarks, generalised Procrustes' analysis (Gower, 1975) was performed. This was implemented using the 'gpagen' function in the *geomorph* R package (Adams & Otárola-Castillo, 2013; Adams *et al*. 2019) to centre, scale and rotate the 2D semi-landmark coordinate data. The Procrustes' superimposed 2D semi-landmarks were used to produce a principal components analysis using the '*plotTangentSpace*' function in the *geomorph* R package (Adams & Otárola-Castillo, 2013; Adams *et al*. 2019). This PCA was used to analyse the distribution of specimens, based on suture shape, in a multivariate morphospace for comparison to suture complexity metrics. Moreover, the PCA was used to identify the major axes of shape variation captured by the sample dataset and whether these reflected variations in complexity.

Complexity analysis

The five methods to determine suture complexity (sinuosity index [SI], suture complexity index [SCI], fractal dimension box counting method [FD box counting], fractal dimension madogram method [FD madogram], short-time Fourier transform with power spectrum density [STFT with PSD]) were applied to the Procrustes superimposed 2D semi-landmarks of the sample dataset (n=79). The methods applied used a range of approaches to capture complexity, with SI and SCI focussing on a linear length approach, fractal dimension employing a statistical index of complexity, and PSD being calculated on Fourier-based method to capture periodic signals using the sum of trigonometric functions. All methods generated a single value indicative of complexity, with low complexity reflected by a low value and higher levels of complexity reflected by a higher value.

Sinuosity index calculations were implemented in base R (v.3.6.0; R Core Team, 2019) using a standard distance between two points equation (Equation 7) in order complete the SI equation (Equation 1, figure 2) (Westerman, 1971). Suture complexity index utilised the SI value calculated in base R (v.3.6.0; R Core Team, 2019) and a complexity factor multiplier which was calculated from the interdigitation lobes (major and minor), as outlined by Saunders (1995) (figure 2). Fractal dimension was calculated in the *fractaldim* R package (Sevcikova *et al.* 2014), using both the box counting and madogram methods by implementing the '*fd.estim.boxcount'* and '*fd.estim.madogram*' functions respectively. STFT was computed using the '*stft*' function in the *e1071* R package (Meyer *et al.* 2019). The PSD of each suture was calculated using the STFT results, by averaging the squared STFT coefficients over each frequency across the local transforms and summing the averages at each harmonic, as described by Allen (2006).

$$D = \sqrt{dx^2 + dy^2}, \qquad \textbf{\textit{Equation 7}}$$

where dx is the distance between the x-coordinates and dy is the distance between the y-coordinates

Comparative analysis of the complexity metrics

Subsequent to the implementation of the five complexity methods (SI, SCI, FD box, FD madogram, and PSD), the appropriateness and effectivity of each metric was assessed using multiple comparative approaches. In order to identify which of the metrics captured the functionally relevant suture morphologies and the variation in complexity, comparisons were made between the shape data and complexity scores. From the PCA produced using the 2D semi-landmark data, PC scores for each PC axis capturing >5% variation were extracted for every specimen. Correspondence between complexity score and PC score were quantified with Pearson's correlation coefficient (r) and visualised with a heatmap using the '*corrplot*' function from the *corrplot* R package (Wei *et al.* 2017). The significance (p<0.05) of the correlations were tested using the '*cor.mtest*' function (Wei *et al.* 2017). To further determine whether the complexity metrics captured the functionally relevant morphological variation of the sample dataset, a heatmap reflecting complexity scores for each specimen was mapped onto the PCA of Procrustes superimposed 2D semi-landmarks.

To assess the similarity among the five different complexity metrics, Pearson's correlation coefficient (r) was quantified and visualised as a heatmap using the '*corrplot*' function in the *corrplot* R package (Wei *et al.* 2017). The significance (p<0.05) of the correlations were tested using the '*cor.mtest*' function (Wei *et al.* 2017).

In order to assess variation and alignment among the methods across our sample, we conducted a further principal components analysis with the specimen complexity scores, rather than shape, obtained by the five complexity methods. The contribution of each variable on the PC axes (i.e. the scaled PC loadings or eigenvectors) were represented on a correlation circle, where each variable equated to one of the five complexity metrics. As each variable trajectory approaches the circle limit, the greater the contribution the method has on the complexity variation explained by the PC axis. The correlation circle was plotted using the *factoextra* R package (Kassambara & Mundt, 2017). A second PCA of complexity was plotted using the '*fviz_pca_biplot*' function in the *factoextra* R package (Kassambara & Mundt, 2017), to plot both specimens (n=79) and variables (n=5) using the complexity scores, in order to summarise the relationships between the methods and specimens and therefore characterise the aspects of complexity captured by the various metric.

Table S1. Specimen details, including the assigned specimen ID.

Specimen ID	Taxon	Specimen Number	Scanning Method
1	*Adapis magnus*	MNHN QU10875	Surface scanned
2	*Adinotherium robustum*	AMNH 9532	Surface scanned
3	*Aepyceros melampus*	NHMUK 66.448	Surface scanned
4	*Agouti paca*	NHMUK 4.12.4.13	Surface scanned
5	*Allodesmus gracilis*	LACM 138167	Surface scanned
6	*Antilocapra americana*	NHMUK 1867.10.5.1 635d	Surface scanned
7	*Aotus trivirgatus*	NHMUK 1976.544	Surface scanned
8	*Aplodontia rufa*	NHMUK 73.1788	CT scanned
9	*Archaelurus debilis*	AMNH 6930	Surface scanned
10	*Archaeohyrax patagonicus*	MACNA_12609a	Surface scanned
11	*Archaeolemur edwardsi*	MNHN MAD8902	Surface scanned
12	*Archaeotherium mortoni*	YPM PU56804	Surface scanned
13	*Arctodyctis sinclairi*	MLP 85-VII-3-1	Surface scanned
14	*Arsinoitherium zitelli*	NHMUK M8463	Surface scanned
15	*Ateles belzebuth*	NHMUK 1854.12.11.1	Surface scanned
16	*Balaena mysticetus*	NHMUK 1986.1.16	Surface scanned
17	*Balaenoptera musculus*	NHMUK 1892.3.1.1	Surface scanned
18	*Borhyaena tubercata*	YPM PU15701	Surface scanned
64	*Brachyuromys betsi*	MNHN 1972N586	CT scanned
19	*Cainotherium laticurvatum*	MNHN SG13456	Surface scanned
20	*Calomyscus baluchi*	NHMUK 69.251	CT scanned
21	*Canis dirus*	NHMUK M11960	Surface scanned
22	*Caperea marginata*	NHMUK 1876.2.16.1	Surface scanned
23	*Capricornis sumatrensis*	NHMUK 24.5.29.1	Surface scanned
24	*Castoroides ohioensis*	NHMUK M7226	Surface scanned
25	*Cavia australis*	NHMUK 26101187	CT scanned
26	*Cervus elaphus*	NHMUK 2005.16	Surface scanned
27	*Chaeropus ecaudatus*	NHMUK 48.1.27.41	Surface scanned
28	*Cheirogaleus medius*	MNHN CG1986_430	Surface scanned
29	*Clyomys laticeps*	NHMUK no specimen number	CT scanned
30	*Cormohipparion occidentale*	AMNH 141219	Surface scanned
31	*Cricetomys gambianus*	CBGP KB36336	CT scanned
32	*Cricetomys gambianus*	CBGP 1977	CT scanned
33	*Ctenomys latro*	NHMUK no specimen number	CT scanned
34	*Cuniculus paca*	UMN 12912	CT scanned
35	*Dacrytherium sp*	MNHN QU17146	Surface scanned
36	*Dasypus novemictus*	Grant Museum z.134	Surface scanned
39	*Dorudon*	NHMUK no specimen number	Surface scanned
65	*Eliurus myoxi*	MNHN 1982N288	CT scanned
40	*Elotherium sp*	AMNH 571	Surface scanned

41	*Eotheroides libyca*	NHMUK M10910	Surface scanned
42	*Euhapsis ellicottae*	KU 48015	Surface scanned
43	*Glaucomys sabrinus*	UMN 5952	Surface scanned
44	*Hippotragus equinus*	NHMUK 1962.12.4.2	Surface scanned
45	*Homocamelus caninus*	AMNH 25060	Surface scanned
46	*Homotherium sp*	AMNH 95297	Surface scanned
47	*Hyaenodon crucians*	AMNH 1372	Surface scanned
48	*Hyracodon nebraskensis*	FMNH P12011	Surface scanned
49	*Iomys horsfieldii*	NHMUK 711522	CT scanned
50	*Janjucetus hunderi*	NMV P216929	Surface scanned
51	*Kobus ellipsiprymnus*	NHMUK 66.817	Surface scanned
52	*Kogia breviceps*	USNM 22015	Surface scanned
53	*Lagostrophus fasciatus*	NHMUK 6.10.5.15c	Surface scanned
37	*Lamprolithax simulans*	LACM 37858	Surface scanned
54	*Lestodon sp*	MACN 11687	Surface scanned
72	*Libysiren sickenbergi*	NHMUK M45675	Surface scanned
55	*Macrotis lagotis*	NHMUK 6.8.1.326	Surface scanned
56	*Megaladapis edwardsi*	MNHN MAD8772a	Surface scanned
57	*Metoreodon profectus*	FMNH P15857	Surface scanned
58	*Microtus ochrogaster*	UMN 4525	CT scanned
59	*Muntiacus muntjack*	NHMUK 15.3.2.1	Surface scanned
61	*Myocastor coypus*	UMN 2805	CT scanned
62	*Mystromys albicaudatus*	NHMUK 1967N1369	CT scanned
63	*Neoparadoxia cecilialina*	LACM 150000	Surface scanned
66	*Pedetes capensis*	NHMUK N502	CT scanned
67	*Pteronarctos goedertae*	LACM 123883	Surface scanned
60	*Rattus norvegicus*	UMN C0304	CT scanned
68	*Ratufa affinis*	NHMUK 55.1724	Surface scanned
69	*Rhinolophus acuminatus*	AMNH 27381	CT scanned
70	*Saimiri sciureus*	NHMUK 27.11.1.18	Surface scanned
38	*Salpintogulus grassicauda*	NHMUK 751793	CT scanned
71	*Sigmodon hispidus*	UMN 6943	CT scanned
73	*Sthenurus andersoni*	WAM 03.5.5	Surface scanned
74	*Tarsius tarsier*	USNM M8665-12063	CT scanned
75	*Thalassocnus littoralis*	MNHN SAS-1615	Surface scanned
76	*Thryonomys swinderianus*	NHMUK 26.11.24.63	Surface scanned
77	*Thylacinus cynocephalus*	Grant Museum z.88	Surface scanned
78	*Thylacosmilus sp*	FMNH P14531	Surface scanned
79	*Tragelaphus scriptus*	NHMUK 8.1.1.128	Surface scanned

Table S2. Summary of the PC axes cumulatively capturing 99% of the overall variation for the 2D semi-landmark data.

Principal Component	Standard Deviation	Proportion of Variance	Cumulative Variance
PC1	0.1451	0.5974	0.5974
PC2	0.0599	0.1019	0.6993
PC3	0.0588	0.0980	0.7973
PC4	0.0427	0.0517	0.8490
PC5	0.0394	0.0440	0.8930
PC6	0.0263	0.0197	0.9127
PC7	0.0217	0.0134	0.9261
PC8	0.0208	0.0123	0.9384
PC9	0.0190	0.0102	0.9485
PC10	0.0174	0.0086	0.9571
PC11	0.0162	0.0075	0.9646
PC12	0.0158	0.0070	0.9717
PC13	0.0123	0.0043	0.9760
PC14	0.0107	0.0033	0.9792
PC15	0.0106	0.0032	0.9824
PC16	0.0094	0.0025	0.9848
PC17	0.0084	0.0020	0.9869
PC18	0.0078	0.0017	0.9887
PC19	0.0073	0.0015	0.9902

Table S3: Complexity scores for all five methods (SI, SCI, FD box counting, FD madogram, PSD) for each specimen (n=79).

Species	FD box counting	FD madogram	PSD	SI	SCI
Adapis magnus	1.0761	1.4749	1.4795	1.1020	1.2122
Adinotherium robustum	1.0729	1.4636	1.5094	1.3621	2.8605
Aepyceros melampus	1.0682	1.4780	1.4707	1.1007	1.4309
Agouti paca	1.0810	1.5255	1.6023	1.2750	1.5300
Allodesmus gracilis	1.0657	1.5152	1.4615	1.2878	7.4693
Antilocapra americana	1.1024	1.5081	1.5236	1.2294	2.7046
Aotus trivirgatus	1.1198	1.6102	1.4904	4.5109	87.9627
Aplodontia rufa	1.0959	1.5860	1.5878	2.1940	21.5011
Archaelurus debilis	1.0650	1.4608	1.4610	1.0478	0.6287
Archaeohyrax patagonicus	1.0997	1.5328	1.5059	1.3614	8.9851
Archaeolemur edwardsi	1.1001	1.4886	1.4757	1.1001	0.7701
Archaeotherium mortoni	1.0942	1.4815	1.4866	1.0922	1.2014
Arctodyctis sinclairi	1.0780	1.5063	1.4969	1.4529	7.8458
Arsinoitherium zitelli	1.0932	1.4671	1.4724	1.1078	0.7754
Ateles belzebuth	1.0809	1.4697	1.4974	1.2761	5.3595
Balaena mysticetus	1.0830	1.4658	1.4623	1.0690	0.8552
Balaenoptera musculus	1.0678	1.4643	1.4598	1.0243	0.2049
Borhyaena tubercata	1.0889	1.4999	1.4966	1.2202	7.4431
Brachyuromys betsi	1.1199	1.6172	1.6753	2.1599	16.1991
Cainotherium laticurvatum	1.0607	1.4647	1.4628	1.0614	1.5921
Calomyscus baluchi	1.0894	1.4916	1.5175	1.1443	2.1742
Canis dirus	1.0994	1.4711	1.4558	1.0983	2.1965
Caperea marginata	1.0991	1.5001	1.4847	1.1144	3.4547
Capricornis sumatrensis	1.0789	1.4561	1.4631	1.0687	0.4275
Castoroides ohioensis	1.0919	1.4919	1.5340	1.6225	13.1425
Cavia australis	1.0795	1.5175	1.5063	1.4623	9.6511
Cervus elaphus	1.1023	1.4767	1.4772	1.0955	0.8764

Chaeropus ecaudatus	1.0829	1.5072	1.6229	1.3339	2.5344
Cheirogaleus medius	1.0813	1.5298	1.6126	1.6120	11.2839
Clyomys laticeps	1.0885	1.5952	1.6700	1.5926	4.1407
Cormohipparion occidentale	1.0992	1.4521	1.4489	1.1413	2.6251
Cricetomys gambianus	1.0994	1.6181	1.7861	4.8609	70.4830
Cricetomys gambianus	1.0991	1.5275	1.5490	2.0364	22.1966
Ctenomys latro	1.0726	1.4764	1.4649	1.1012	1.3214
Cuniculus_paca	1.0735	1.4700	1.4748	1.1343	3.0627
Dacrytherium sp	1.0799	1.5881	1.6281	1.3563	2.5770
Dasypus novemictus	1.0956	1.5451	1.5231	1.4261	6.2748
Dorudon	1.0660	1.4615	1.4560	1.0537	1.1591
Eliurus myoxi	1.0872	1.5238	1.5600	1.3742	4.8096
Elotherium sp	1.0907	1.4630	1.4553	1.0654	1.1720
Eotheroides libyca	1.0934	1.5139	1.4974	1.1918	2.2645
Euhapsis ellicottae	1.0952	1.4770	1.4993	1.1774	1.5306
Glaucomys sabrinus	1.0954	1.5472	1.4994	1.3341	6.5372
Hippotragus equinus	1.0938	1.4662	1.4612	1.0782	1.2938
Homocamelus caninus	1.0776	1.5146	1.5001	1.2246	2.6940
Homotherium sp	1.1187	1.6565	1.9275	2.0583	4.9398
Hyaenodon crucians	1.0884	1.4747	1.4618	1.0682	1.3887
Hyracodon nebraskensis	1.0688	1.4631	1.4579	1.0442	1.2531
Iomys horsfieldii	1.1313	1.6105	1.6967	2.4388	25.6072
Janjucetus hunderi	1.0802	1.5165	1.4660	1.2545	5.3942
Kobus ellipsiprymnus	1.0624	1.4639	1.4634	1.0447	0.8358
Kogia breviceps	1.0548	1.4919	1.4690	1.0649	1.4909
Lagostrophus fasciatus	1.0754	1.522611803	1.5908	1.5395	6.7739
Lamprolithax simulans	1.0951	1.4607	1.4765	1.0411	0.5205
Lestodon sp	1.0711	1.5470	1.6895	1.6359	6.8710
Libysiren sickenbergi	1.0628	1.5409	1.5506	1.4865	4.7568
Macrotis lagotis	1.0927	1.4731	1.5116	1.0853	0.4341
Megaladapis edwardsi	1.0907	1.4986	1.4941	1.3030	4.5607

Metoreodon profectus	1.0851	1.4786	1.4845	1.1086	2.3280
Microtus ochrogaster	1.0827	1.4836	1.4621	1.1317	1.8108
Muntiacus muntjack	1.0747	1.5181	1.5214	1.2593	3.1482
Myocastor coypus	1.0734	1.4824	1.4586	1.4753	4.5734
Mystromys albicaudatus	1.0634	1.5024	1.6703	1.7360	7.6385
Neoparadoxia cecilialina	1.0864	1.4915	1.5272	1.1591	3.0136
Pedetes capensis	1.0831	1.5727	1.6170	1.6500	6.6000
Pteronarctos goedertae	1.0797	1.5592	1.6600	1.5174	5.7660
Rattus norvegicus	1.0987	1.5573	1.6005	2.0598	11.3289
Ratufa affinis	1.0764	1.5158	1.4647	1.3864	5.5456
Rhinolophus acuminatus	1.0937	1.4821	1.4844	1.1889	2.1400
Saimiri sciureus	1.1037	1.5314	1.4901	1.2041	3.2512
Salpintogulus grassicauda	1.0702	1.4769	1.4652	1.1469	3.4408
Sigmodon hispidus	1.1027	1.4939	1.5372	2.0006	17.8055
Sthenurus andersoni	1.0681	1.4591	1.4600	1.0360	1.0360
Tarsius tarsier	1.0755	1.5117	1.4832	1.2486	6.3677
Thalassocnus littoralis	1.0851	1.4635	1.4761	1.2006	1.9209
Thryonomys swinderianus	1.0923	1.5692	1.5571	1.3836	4.5660
Thylacinus cynocephalus	1.0990	1.6265	1.8218	1.9988	6.7935
Thylacosmilus sp	1.0904	1.4590	1.4609	1.0385	0.8308
Tragelaphus scriptus	1.0685	1.4603	1.4619	1.0450	1.4630

Table S4. Specimen PC scores for the PC axes that each explain >5% overall variation in suture shape from 2D semi-landmarks.

Species	PC1	PC2	PC3	PC4
Adapis magnus	-0.0751	0.0217	-0.0100	0.0055
Adinotherium robustum	-0.1607	0.0304	0.1478	0.0257
Aepyceros melampus	0.0456	0.0057	-0.0045	0.0229
Agouti paca	-0.2323	-0.0046	-0.0372	-0.0032
Allodesmus gracilis	-0.0079	0.0293	0.0256	0.0443
Antilocapra americana	-0.0257	-0.0799	0.0063	0.0138
Aotus trivirgatus	-0.0722	0.0227	0.0044	-0.0568
Aplodontia rufa	0.2277	-0.0094	-0.0836	0.0987
Archaelurus debilis	0.0127	0.0154	-0.0048	0.0054
Archaeohyrax patagonicus	0.0188	0.0068	-0.1117	-0.0320
Archaeolemur edwardsi	0.0385	0.0287	-0.0520	-0.0207
Archaeotherium mortoni	-0.0798	0.0113	-0.0145	-0.0258
Arctodyctis sinclairi	0.0404	0.0532	-0.1104	0.0343
Arsinoitherium zitelli	-0.0468	-0.0001	0.0268	0.0021
Ateles belzebuth	-0.1240	0.0318	0.0297	0.0259
Balaena mysticetus	-0.0453	0.0108	0.0382	0.0063
Balaenoptera musculus	0.0390	0.0238	-0.0171	0.0088
Borhyaena tubercata	-0.0766	0.0106	-0.0366	0.0331
Brachyuromys betsi	0.0215	-0.1360	-0.1652	-0.0728
Cainotherium laticurvatum	0.0026	0.0066	0.0005	0.0096
Calomyscus baluchi	-0.1118	0.0141	-0.0447	-0.0187
Canis dirus	0.0157	0.0151	0.0217	0.0017
Caperea marginata	0.1013	0.0226	-0.0247	0.0095
Capricornis sumatrensis	0.0147	0.0165	-0.0052	0.0037
Castoroides ohioensis	-0.0551	0.0706	-0.0124	-0.0113
Cavia australis	-0.0947	-0.0093	0.1482	-0.0008
Cervus elaphus	0.0318	-0.0106	0.0042	0.0169
Chaeropus ecaudatus	-0.2064	-0.0500	0.0114	-0.0477
Cheirogaleus medius	-0.2607	-0.0208	0.0418	-0.0505
Clyomys laticeps	0.3247	-0.0128	-0.0600	0.0499
Cormohipparion occidentale	0.0397	0.0511	0.0303	0.0211
Cricetomys gambianus	-0.3369	-0.0963	-0.0190	0.1756
Cricetomys gambianus	-0.0182	-0.0599	-0.0341	-0.0144
Ctenomys latro	0.0104	0.0193	-0.0101	0.0319
Cuniculus paca	-0.0760	0.0204	0.0353	-0.0277
Dacrytherium sp	0.3285	0.0088	-0.0513	0.0249
Dasypus novemictus	0.2153	0.0667	0.1203	0.0258
Dorudon	0.0191	0.0146	-0.0005	0.0042
Eliurus myoxi	0.0498	0.0646	-0.0987	-0.1122
Elotherium sp	-0.0165	0.0242	0.0196	0.0127
Eotheroides libyca	0.1186	0.0084	-0.0324	0.0288
Euhapsis ellicottae	-0.0136	-0.0161	0.0067	-0.0063
Glaucomys sabrinus	0.1726	0.0580	-0.0879	0.0140
Hippotragus equinus	0.0028	0.0305	-0.0241	0.0040
Homocamelus caninus	0.1656	0.0547	-0.0022	-0.0289
Homotherium sp	0.1635	-0.3484	-0.0292	-0.0280
Hyaenodon crucians	0.0394	0.0390	-0.0385	0.0055

Hyracodon nebraskensis	0.0122	0.0200	0.0107	0.0169
Iomys horsfieldii	-0.2760	-0.1357	-0.0144	0.0090
Janjucetus hunderi	-0.0095	-0.0074	0.0198	0.0632
Kobus ellipsiprymnus	0.0155	0.0170	-0.0192	0.0133
Kogia breviceps	0.0944	0.0271	-0.0037	-0.0228
Lagostrophus fasciatus	-0.2320	0.0075	0.0009	-0.0246
Lamprolithax simulans	0.0185	0.0072	-0.0190	-0.0084
Lestodon sp	-0.2875	-0.0100	-0.0553	-0.0089
Libysiren sickenbergi	0.1572	-0.0108	0.0417	-0.0785
Macrotis lagotis	-0.0758	-0.0011	-0.0205	-0.0350
Megaladapis edwardsi	-0.0057	0.0600	-0.0175	-0.0687
Metoreodon profectus	-0.0924	0.0071	0.0093	-0.0110
Microtus ochrogaster	0.0261	0.0054	0.0037	0.0109
Muntiacus muntjack	-0.0595	0.0222	0.0521	-0.1308
Myocastor coypus	-0.0173	0.0373	-0.0121	0.0076
Mystromys albicaudatus	-0.2854	-0.0405	0.0660	-0.0564
Neoparadoxia cecilialina	-0.1102	-0.0094	-0.0408	-0.0353
Pedetes capensis	0.2918	0.0447	0.1987	-0.0264
Pteronarctos goedertae	-0.3529	0.0293	0.0254	0.0799
Rattus norvegicus	0.1944	-0.0392	-0.0603	0.0078
Ratufa affinis	0.1256	0.0628	-0.0316	0.0719
Rhinolophus acuminatus	-0.0042	0.0296	-0.0226	-0.0015
Saimiri sciureus	0.1467	-0.0091	0.0394	0.0171
Salpintogulus grassicauda	0.0017	-0.0076	0.0919	0.0105
Sigmodon hispidus	-0.0259	-0.0285	-0.0058	0.0316
Sthenurus andersoni	0.0188	0.0126	-0.0015	0.0099
Tarsius tarsier	0.1161	0.0447	0.0191	-0.0355
Thalassocnus littoralis	-0.0881	0.0159	0.0454	0.0069
Thryonomys swinderianus	0.2248	-0.0378	0.0211	-0.0434
Thylacinus cynocephalus	0.3329	-0.1964	0.1760	0.0123
Thylacosmilus sp	-0.0055	0.0128	0.0136	-0.0009
Tragelaphus scriptus	0.0271	0.0161	-0.0070	0.0101

Table S5. Correlation coefficients for the correlations between the complexity scores of the five tested methods (SI, SCI, FD box counting, FD madogram, PSD) and the PC scores from the PCA of 2D semi-landmarks for the PC axes accounting for >5% of the overall variation.

	PC1	PC2	PC3	PC4
**FD box counting**	0.0731	- 0.4498	- 0.2224	- 0.0009
**FD madogram**	0.2102	- 0.5924	- 0.0902	0.0410
**PSD**	- 0.0544	- 0.7660	0.0037	0.0264
**SI**	- 0.1758	- 0.3836	- 0.0545	0.2064
**SCI**	- 0.2170	- 0.1940	- 0.0743	0.1792

Table S6. Correlation coefficients for the correlations between the five tested methods (SI, SCI, FD box counting, FD madogram, PSD).

	FD box counting	FD madogram	PSD	SI	SCI
**FD box counting**	1.0000	0.4881	0.3553	0.4467	0.4102
**FD madogram**	0.4881	1.0000	0.8234	0.6889	0.5335
**PSD**	0.3553	0.8234	1.0000	0.5535	0.3138
**SI**	0.4467	0.6889	0.5535	1.0000	0.9535
**SCI**	0.4102	0.5335	0.3138	0.9535	1.0000

Table S7. Summary of axes for the principal components analysis of complexity scores.

	PC1	PC2	PC3	PC4	PC5
**Eigenvalue**	3.2689	0.8897	0.6843	0.1470	0.0101
**Projected inertia (%)**	65.3781	17.7939	13.6869	2.9389	0.2022
**Cumulative projected inertia (%)**	65.38	83.17	96.86	99.80	100.00

Figure S1. Two-dimensional suture landmarks digitsed in the *Stereomorph* R package (Olsen & Haber, 2018), for specimens: *Adapis magnus; Adinotherium robustum; Aepyceros melampus; Agouti paca; Allodesmus gracillis; Antilocapra Americana; Aoutus trivirgatus; Aplodontia rufa*.

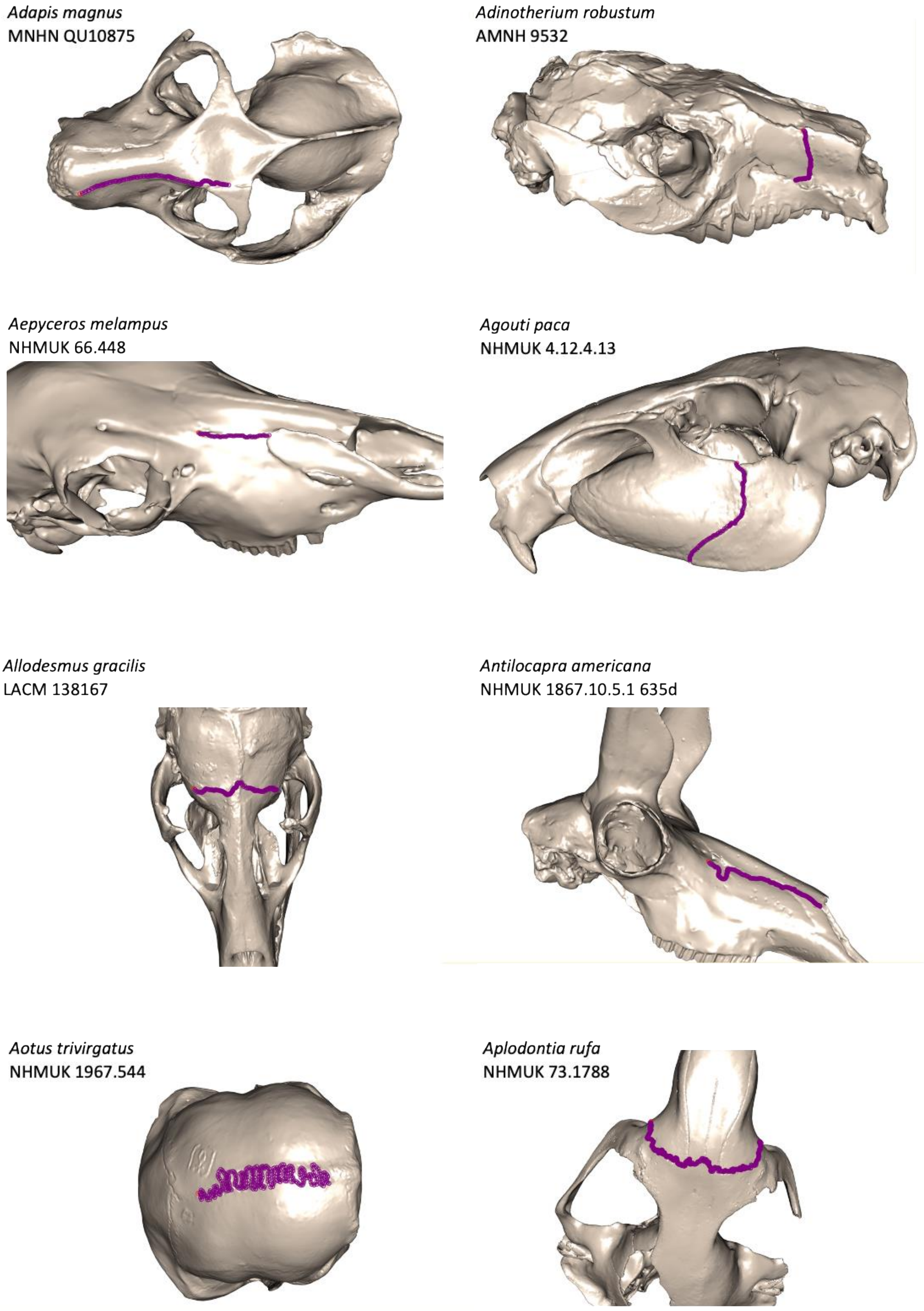

Figure S1 continued. Two-dimensional suture landmarks digitised in the *Stereomorph* R package (Olsen & Haber, 2018), for specimens: *Archaelurus debilis; Archaehyrax patagonicus; Archaeolemur edwardsi; Archaeotherium mortoni; Arctodyctis sinclairi; Arsinoitherium zitelli; Ateles belzebuth; Balaena mysticetus.*

Archaelurus debilis
AMNH 6930

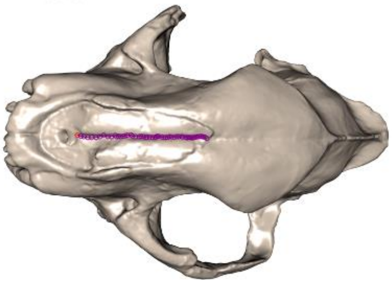

Archaehyrax patagonicus
MACNA_12609a

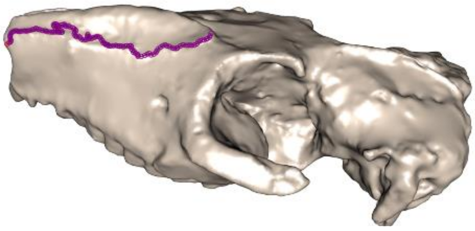

Archaeolemur edwardsi
MNHN MAD 8902

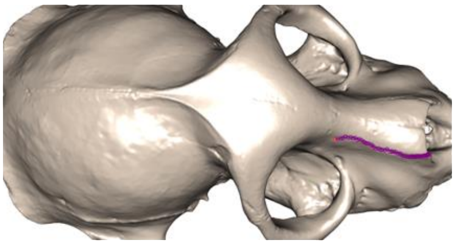

Archaeotherium mortoni
YPM PU56804

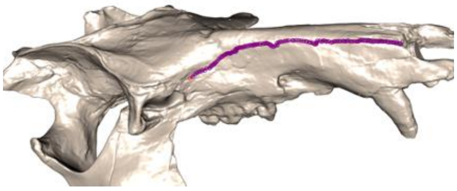

Arctodyctis sinclairi
MLP 85-VII-3-1

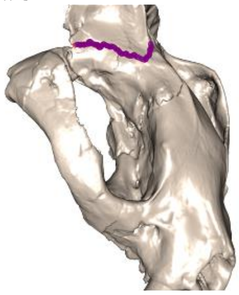

Arsinoitherium zitelli
NHMUK M8463

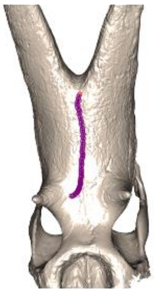

Ateles belzebuth
NHMUK 1854.12.11.1

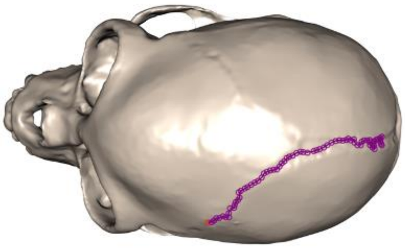

Balaena mysticetus
NHMUK 1986.1.16

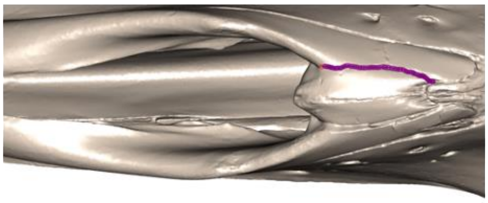

Figure S1 continued. Two-dimensional suture landmarks digitised in the *Stereomorph* R package (Olsen & Haber, 2018), for specimens: *Balaenoptera musculus; Borhyaena tubercata; Brachyuromys betsi; Cainotherium laticurvatum; Calomyscus baluchi; Canis dirus; Caperea marginata; Capricornis sumatrensis.*

Balaenoptera musculus
NHMUK 1892.3.1.1

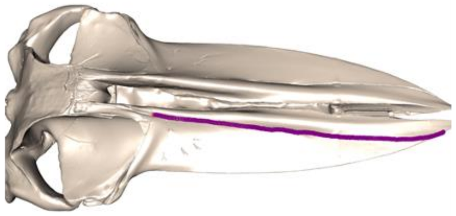

Borhyaena tubercata
YPM PU15701

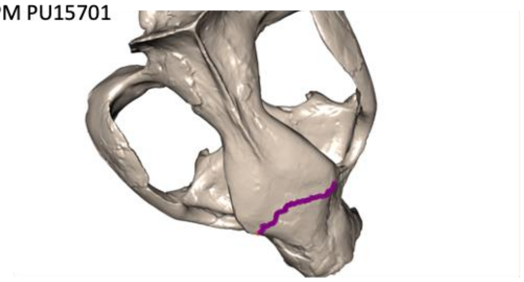

Brachyuromys betsi
MNHN 1972N586

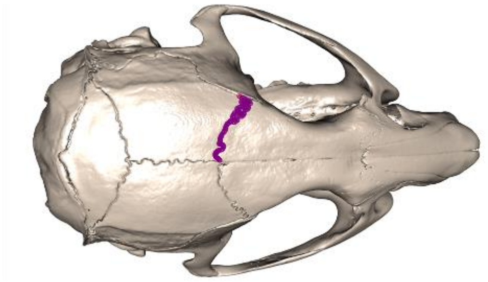

Cainotherium laticurvatum
MNHN SG13456

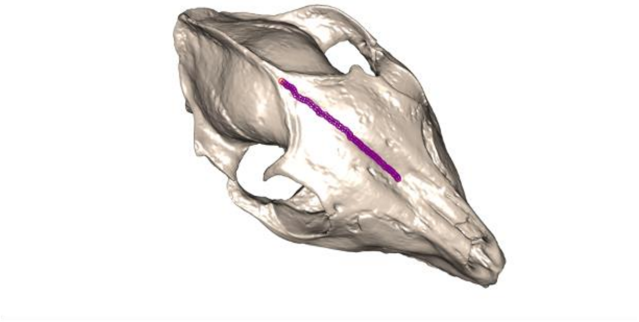

Calomyscus baluchi
NHMUK 69.251

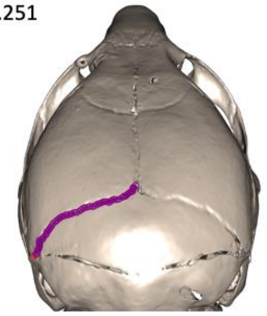

Canis dirus
NHMUK M11960

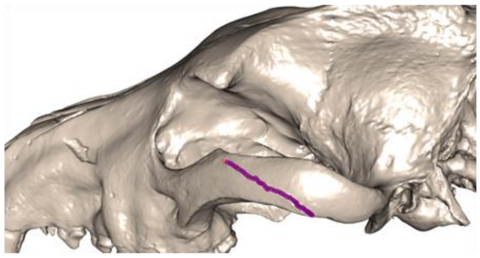

Caperea marginata
NHMUK 1876.2.16.1

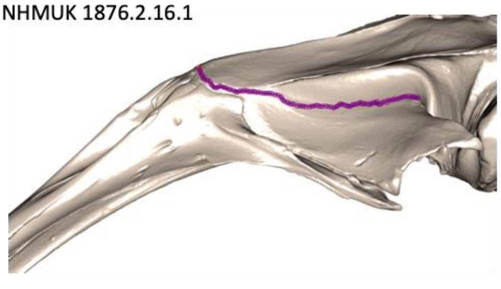

Capricornis sumatrensis
NHMUK 24.5.29.1

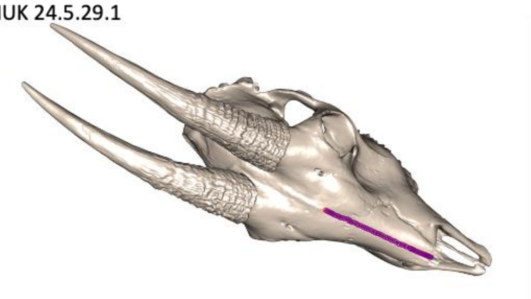

Figure S1 continued. Two-dimensional suture landmarks digitised in the *Stereomorph* R package (Olsen & Haber, 2018), for specimens: *Castoroides ohioensis; Cavia australis; Cervus elaphus; Chaeropus ecaudatus; Cheirogaleus medius; Clyomys laticeps; Cormohipparion occidentale; Cricetomys gambianus.*

Castoroides ohioensis
NHMUK M7226

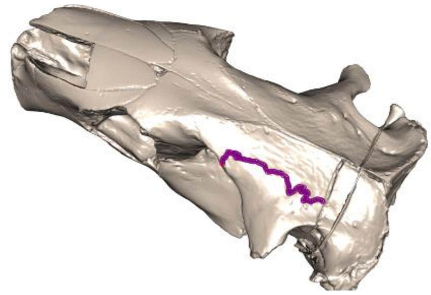

Cavia australis
NHMUK 26101187

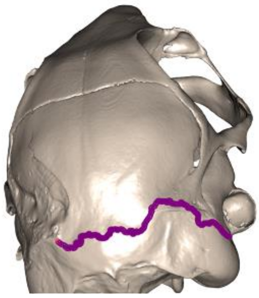

Cervus elaphus
NHMUK 2005.16

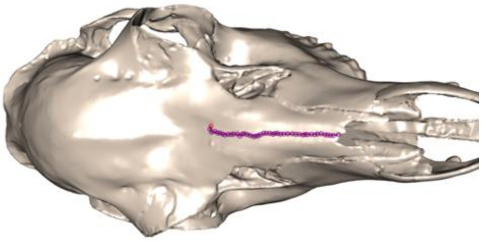

Chaeropus ecaudatus
NHMUK 48.1.27.41

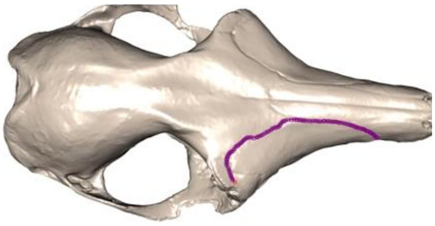

Cheirogaleus medius
MNHN CG1986_430

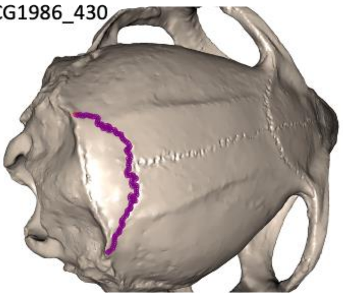

Clyomys laticeps
NHMUK no specimen number

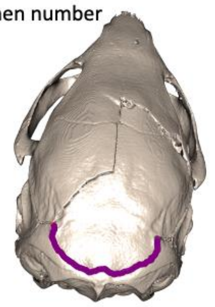

Cormohipparion occidentale
AMNH 141219

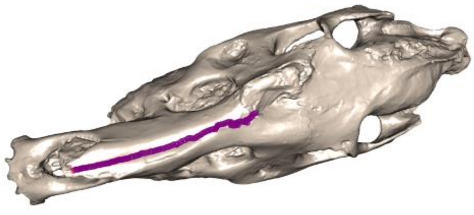

Cricetomys gambianus
NHMUK 34.4.1.158

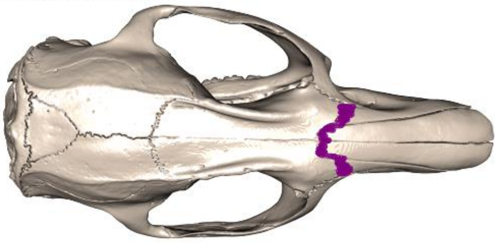

Figure S1 continued. Two-dimensional suture landmarks digitised in the *Stereomorph* R package (Olsen & Haber, 2018), for specimens: *Cricetomys gambianus; Ctenomys latro; Cuniculus paca; Dacrytherium sp; Dasypus novemictus; Dorudon; Eliurus myoxi; Elotherium sp.*

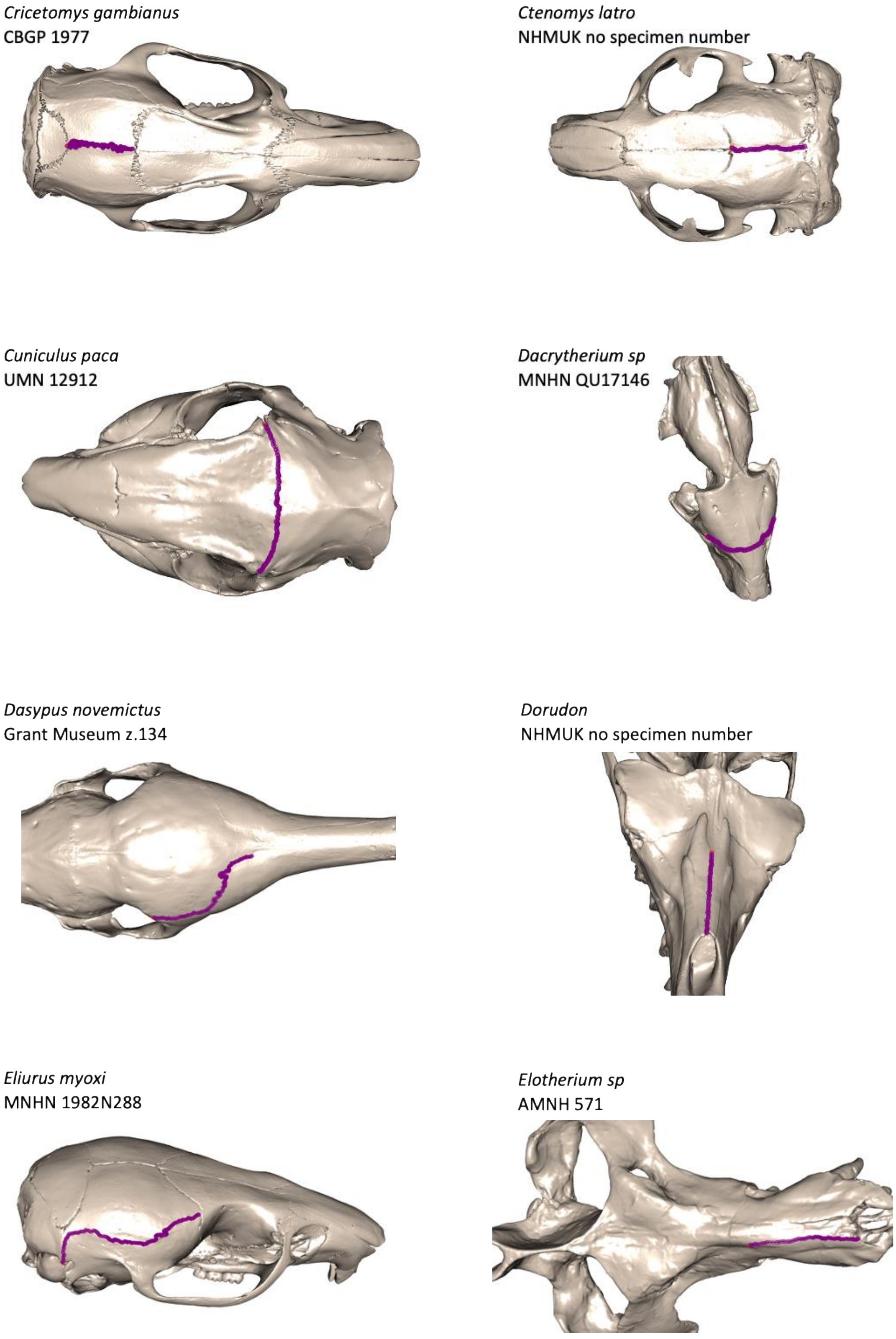

Figure S1 continued. Two-dimensional suture landmarks digitised in the *Stereomorph* R package (Olsen & Haber, 2018), for specimens: *Eotheroides libyca; Euhapsis ellicottae; Glaucomys sabrinus; Hippotragus equinus; Homocamelus caninus; Homotherium sp; Hyaenodon crucians; Hyracodon nebraskensis.*

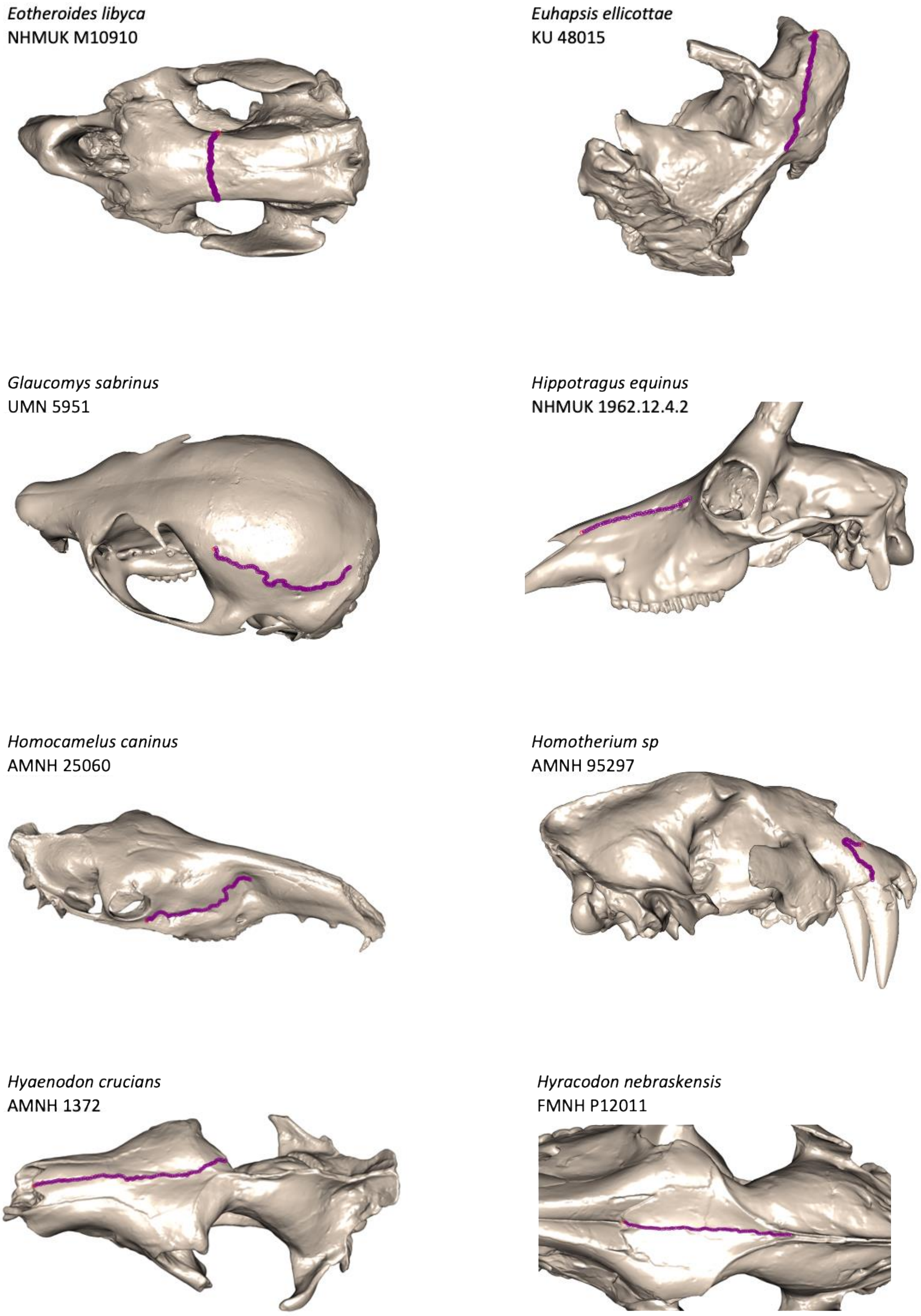

Figure S1 continued. Two-dimensional suture landmarks digitised in the *Stereomorph* R package (Olsen & Haber, 2018), for specimens: *Iomys horsefieldii; Janjucetus hunderi; Kobus ellipsiprymnus; Kogia breviceps; Lagostrophus fasciatus; Lamprolithax simulans; Lestodon sp; Libysiren sickenbergi.*

Iomys horsefieldii
NHMUK 711522

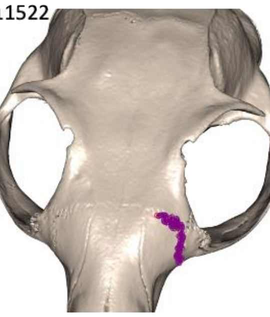

Janjucetus hunderi
NHV P216929

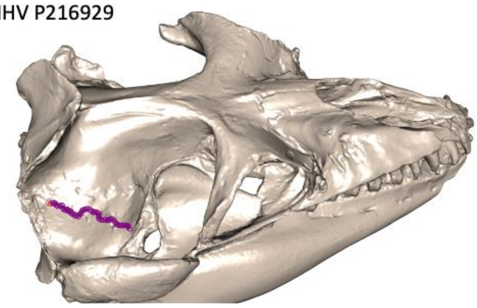

Kobus ellipsiprymnus
NHMUK 66.817

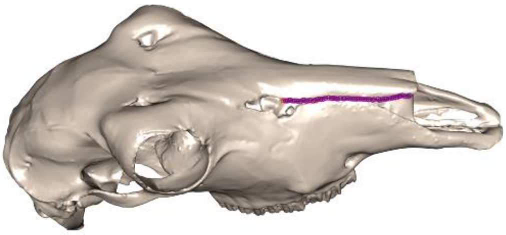

Kogia breviceps
USNM 22015

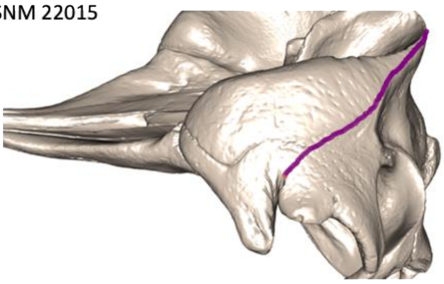

Lagostrophus fasciatus
NHMUK 6.10.5.15c

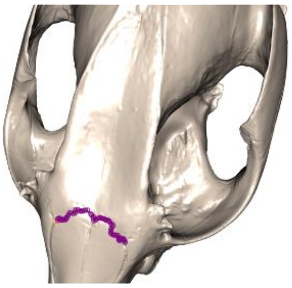

Lamprolithax simulans
LACM 37858

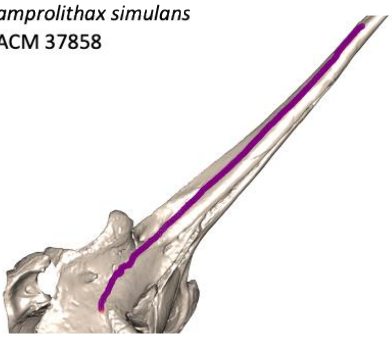

Lestodon sp
MACN 11687

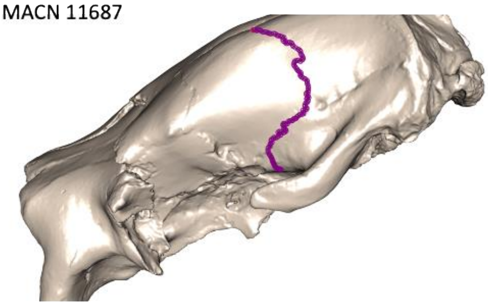

Libysiren sickenbergi
NHMUK M45675

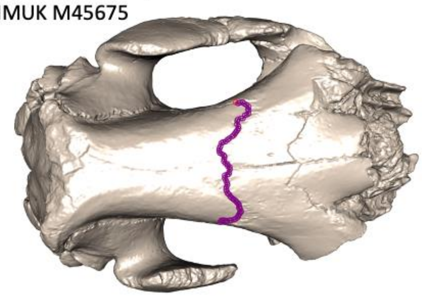

Figure S1 continued. Two-dimensional suture landmarks digitised in the *Stereomorph* R package (Olsen & Haber, 2018), for specimens: *Macrotis lagotis; Megaladapis edwardsi; Metoreodon profectus; Microtus ochragaster; Muntiacus muntjack; Myocastor coypus; Mystromys albicaudatus; Neoparadoxia cecilianlina.*

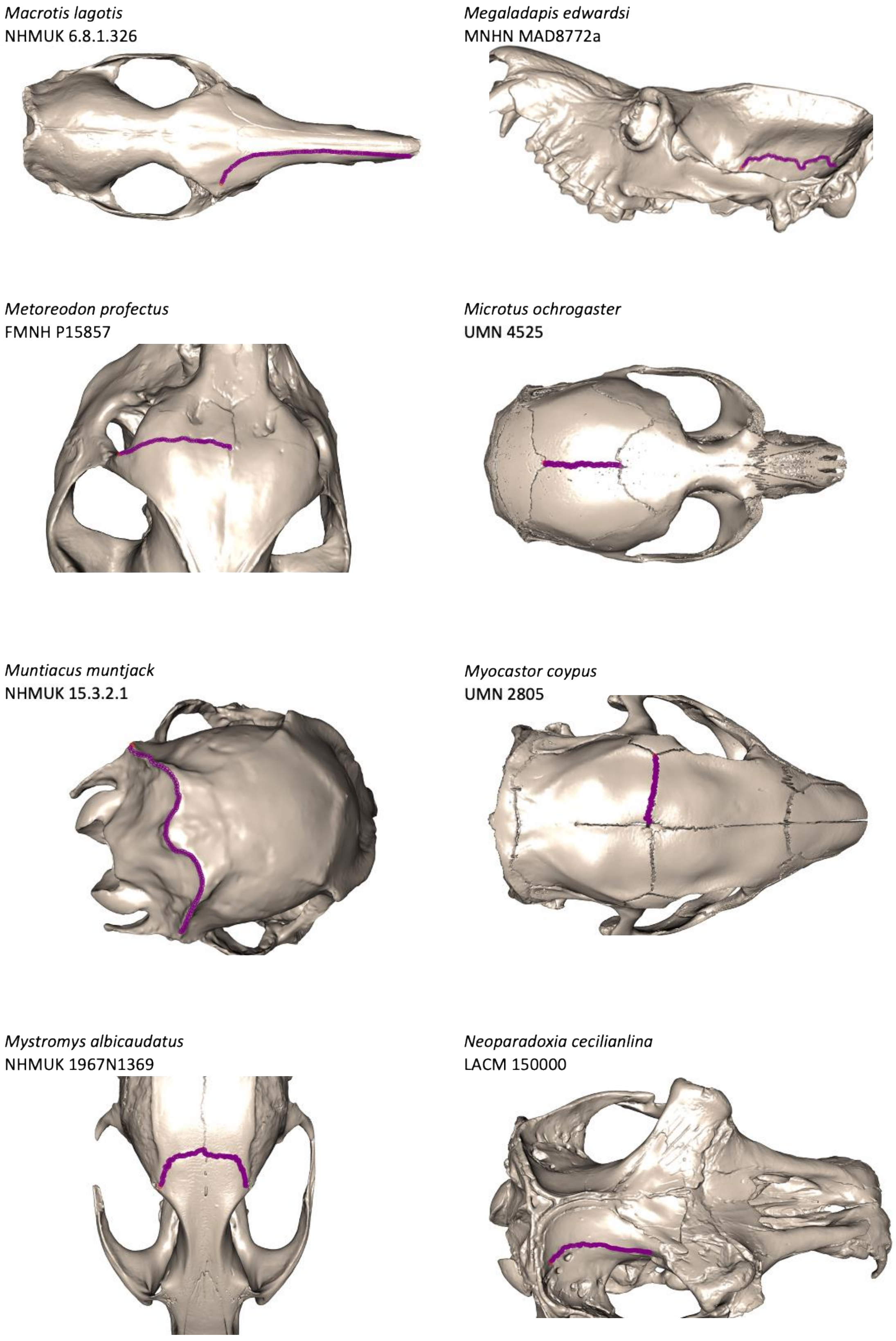

Figure S1 continued. Two-dimensional suture landmarks digitised in the *Stereomorph* R package (Olsen & Haber, 2018), for specimens: *Pedetes capensis; Pteronarctos goedertae; Rattus norvegicus; Ratufa affinis; Rhinolophus acuminatus; Saimiri sciureus; Salpintogulus grassicauda; Sigmodon hispidus.*

Pedetes capensis
NHMUK N502

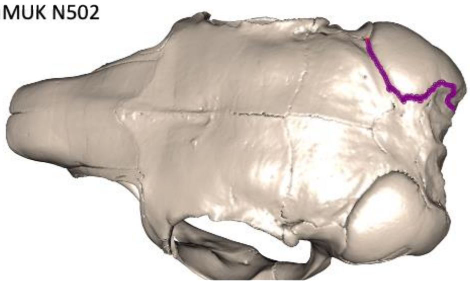

Pteronarctos goedertae
LACM 123883

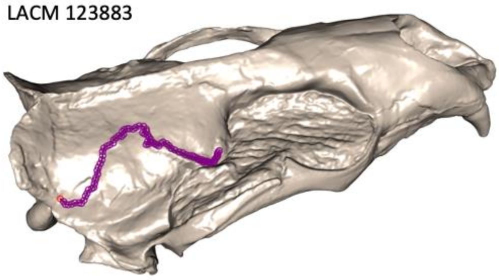

Rattus norvergicus
UMN C0304

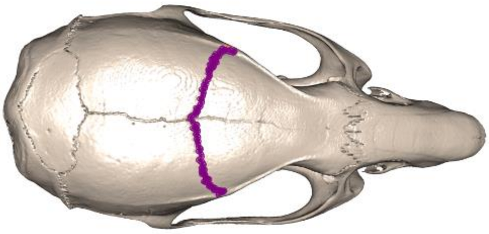

Ratufa affinis
NHMUK 55.1724

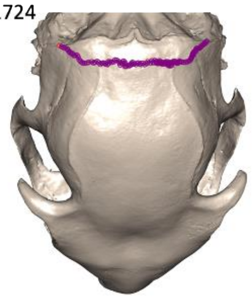

Rhinolophus acuminatus
AMNH 27381

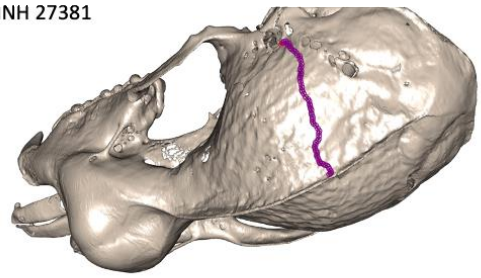

Saimiri sciureus
NHMUK 27.11.1.18

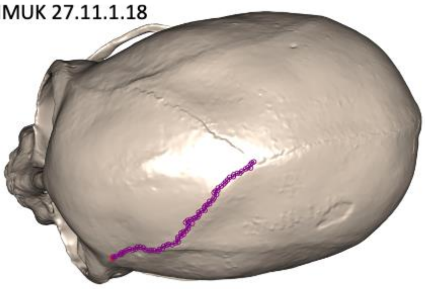

Salpintogulus grassicauda
NHMUK 751793

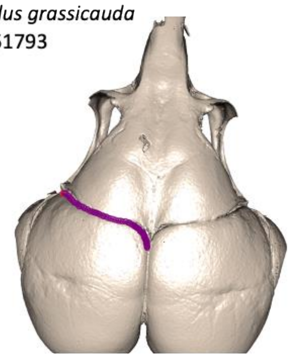

Sigmodon hispidus
UMN 6943

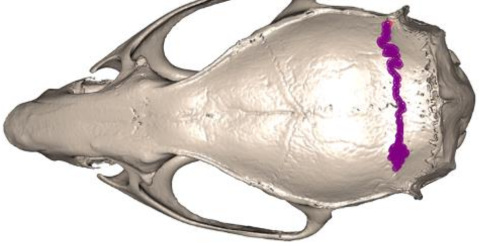

Figure S1 continued. Two-dimensional suture landmarks digitised in the *Stereomorph* R package (Olsen & Haber, 2018), for specimens: *Sthenurus andersoni; Tarsius tarsier; Thalassocnus littoralis; Thryonomys swinderianus; Thylacinus cynocephalus; Thylacosmilus sp; Tragelaphus scriptus.*

Sthenurus andersoni
WAM 03.5.5

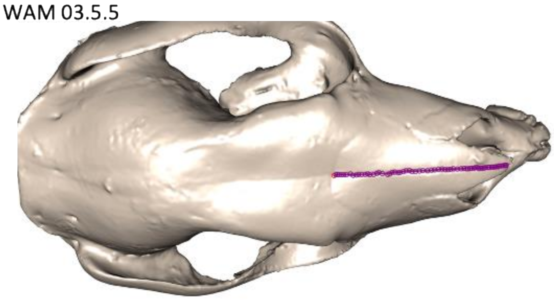

Tarsius tarsier
USNM M8665-12063

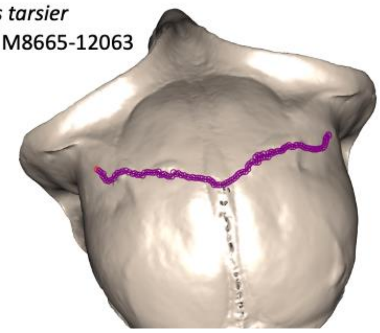

Thalassocnus littoralis
MNHN SAS-1615

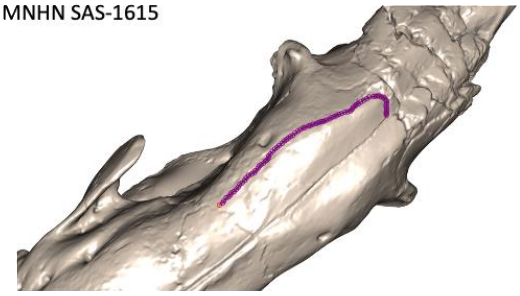

Thryonomys swinderianus
NHNUK 26.11.24.63

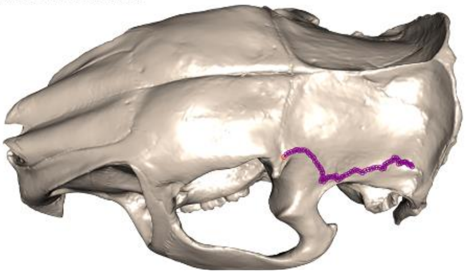

Thylacinus cynocephalus
Grant Museum z.88

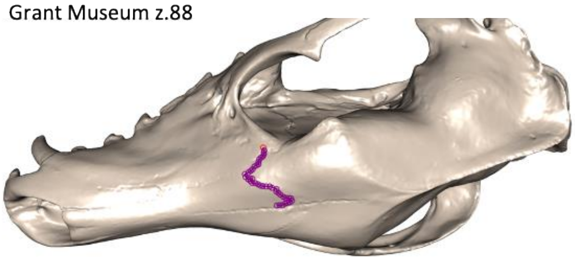

Thylacosmilus sp
FMNH P14531

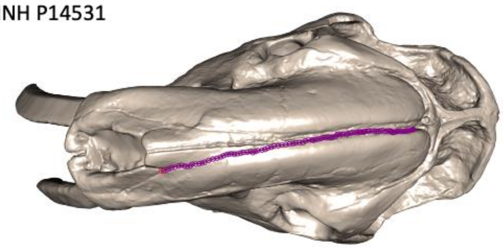

Tragelaphus scriptus
NHMUK 8.1.1.128

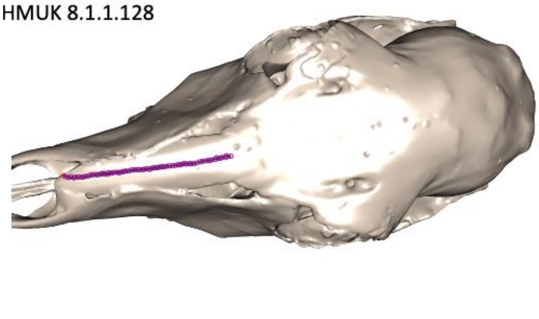

Figure S2. Extreme suture morphologies for the PC axes explaining >5% of the overall variation from PCA of 2D semi-landmarks, with the negative extreme indicated on the left and positive extreme indicated on the right.

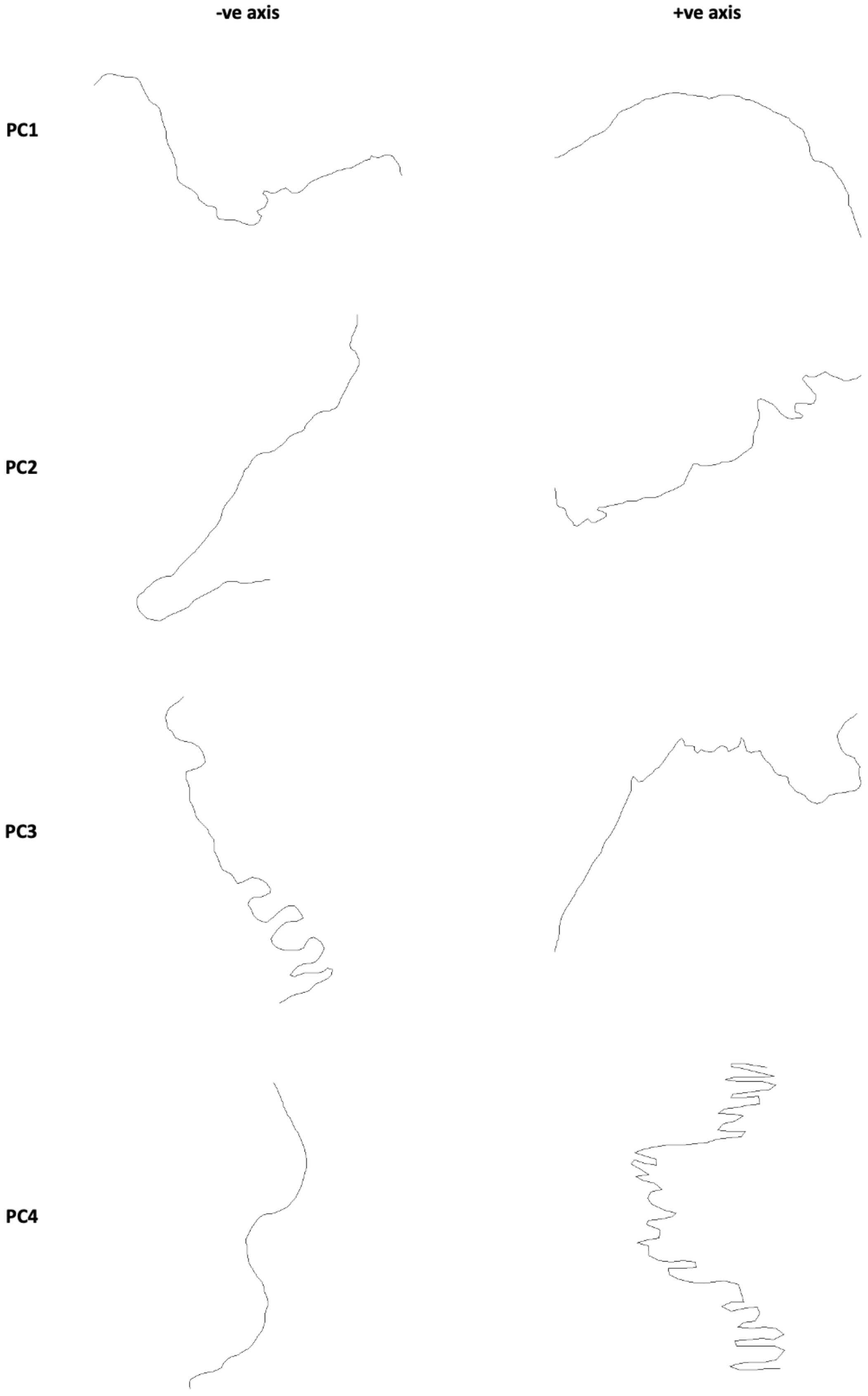

Figure S3. Least and most complex sutures identified by each complexity method (SI, SCI, FD box counting, FD madogram, PSD).

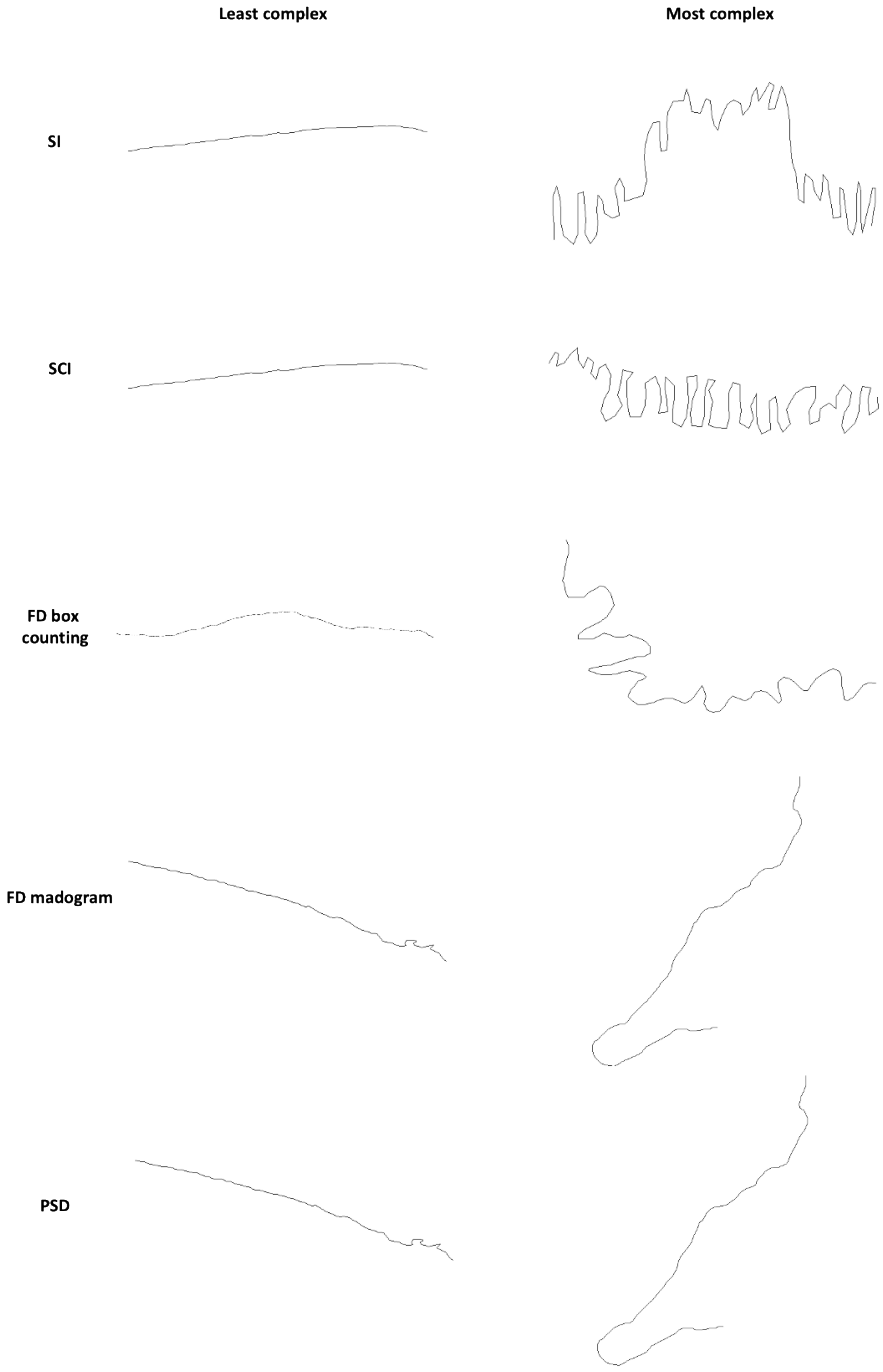

Figure S4. PC loadings for each of the five methods (SI, SCI, FD box counting, FD madogram, PSD) on the PCA of complexity scores, for the PC axes contributing to >5% of the overall variation: (*a*) PC1; (*b*) PC2; (*c*) PC3.

(a)

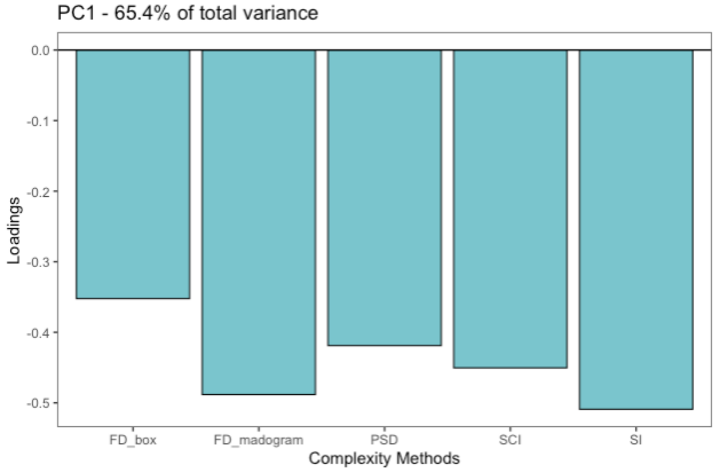

(b)

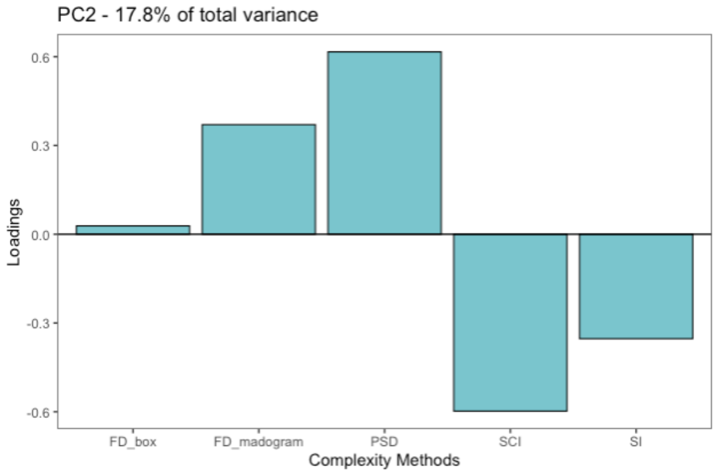

(c)

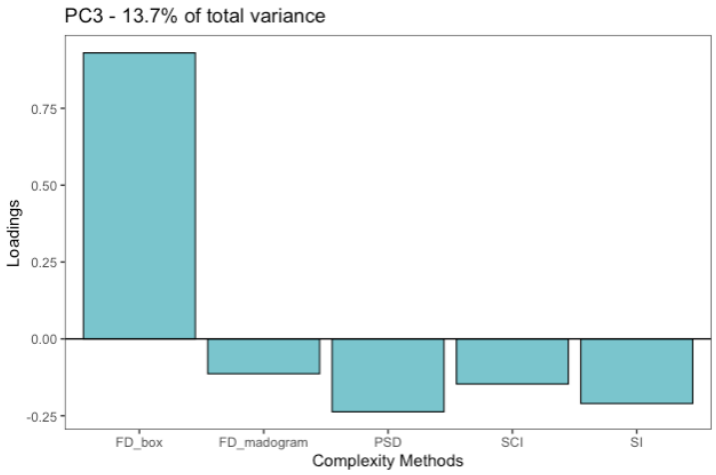

Supplementary Information References

Adams DC, Collyer ML, Kaliontzopoulou A. 2019 *geomorph: Geometric Morphometric Analyses of 2D/3D Landmark Data*. R package version 3.1.3. https://cran.r-project.org/web/packages/geomorph/index.html

Adams DC, Otárola-Castillo E. 2013 Geomorph: An R package for the collection and analysis of geometric morphometric shape data. *Methods Ecol. Evol.* **4**, 393-399

Adler D, Murdoch M. 2019 *rgl: 3D Visualization Using OpenGL*. R package version 0.100.26 https://cran.r-project.org/web/packages/rgl/index.html

Allen EG. 2006 New approaches to Fourier analysis of ammonoid sutures and other complex, open curves. *Paleobiology* **32**, 299-315

Bardua C, Evans SE, Goswami A. 2018 Phylogeny, ecology and deep time: 2D outline analysis of anuran skulls from the Early Cretaceous to the Recent. *Palaeontology* **62**, 417-431

Boyajian G, Lutz T. 1992 Evolution of biological complexity and its relation to taxonomic longevity in the Ammonoidea. *Geology* **20**, 983-986

Carlo JM, Barbeitos MS, Lasker HR. 2011 Quatifying complex shapes: Elliptical Fourier Analysis of octocoral sclerites. *Biol. Bull.* **220**, 224-237

Crampton JS. 1995 Elliptical Fourier shape analysis of fossil bivalves: some practical considerations. *Lethaia* **28**, 179-186

Emmons CK, Hard JJ, Dahlheim ME, Waite JM. 2018 Quantifying variation in killer whale (Orcinus orca) morphology using elliptical Fourier analysis. *Mar. Mam. Sci.* **35**, 5-21

Gildner RF. 2003 A Fourier method to describe and compare suture patterns. *Palaeontol. Electron.* **6**, 1-12

Gneiting T Sevcikova H, Percival DB. 2012 Estimators of fractal dimension: Assessing the smoothness of time series and spatial data. *Stat. Sci.* **27**, 247-277

Gower JC. 1975 Generalized procrustes analysis. *Psychometrika* **40**, 33-51

Kak AC, Slaney M. 1988 *Principles of Computerized Tomographic Imaging*. New York: IEEE Press

Kassambara A, Mundt F. 2017 *factoextra: Extract and Visualize the Results of Multivariate Data Analyses*. R package 1.0.5. https://cran.r-project.org/web/packages/factoextra/index.html

Long CA. 1985 Intricate sutures as fractal curves. *J. Morphol.* **185**, 285-295

Long CA, Long JE. 1992 Fractal dimensions of cranial sutures and waveforms. *Acta. Anat.* **145**, 201-206

Mandelbrot B. 1982 *The fractal geometry of nature*. New York: W H Freeman & Co

Meyer D, Dimitriadou E, Hornik K, Weingessel A, Leisch F, Chang C-C, Lin C-C. 2019 *e1071: Misc Functions of the Department of Statistics, Probability Theory Group (Formerly: E1071), TU Wien*. R package version 1.7.2 https://cran.r-project.org/web/packages/e1071/index.html

Monteiro LR, Lessa LG. 2000 Comparative analysis of cranial suture complexity in the genus Caiman (Crocodylia, Alligatoridae*). Rev. Brasil Biol.* **60**, 689-694

Olsen A, Haber A.2018 *StereoMorph: Stereo Camera Calibration and Reconstruction*. R package version 1.6.2. https://cran.r-project.org/web/packages/StereoMorph/index.html

Rohlf FJ, Slice DE. 1990 Extensions of the Procrustes method for the optimal superimposition of landmarks. *Syst. Zool.* **39**, 40-59

Saunders WB. 1995 The ammonoid suture problem: Relationships between shell and septum thickness and suture complexity in Paleozoic ammonoids. *Paleobiology* **21**, 343-355

Sevcikova H, Percival D, Gneiting T. 2014 *fractaldim: Estimation of fractal dimensions*. R package version 0.8-4 https://cran.r-project.org/web/packages/fractaldim/index.html

Skrzat J, Walocha. 2003 Application of fractal dimension in evaluation of cranial suture complexity. *Harmonic Fractal Image Anal.* **2003**, 39-41

Tort A. 2003 Elliptical Fourier functions as a morphological descriptor of the genus *Stenosarina* (Brachiopoda, Terebratulida) *Math. Geosci.* **35**, 873-885

Wei T, Simko V, Levy M, Xie Y, Jin Y, Zemla J. 2017 corrplot: Visualization of a Correlation Matrix. R package version 0.84 https://cran.r-project.org/web/packages/corrplot/index.html

Westerman GEG. 1971 Form, structure and function of shell and siphuncle in coiled Mesozoic ammonoids. *Life Sci. Contrib. R. Ont. Mus.* **78**, 1-39

Wu YD, Chien CH, Chao YJ, Yu JC, Williamson MA. 2007 Fourier analysis of human sagittal sutures. *Cleft Palate Craniofac. J.* **44**, 482-493
